# Compound loss of muscleblind-like function in myotonic dystrophy

**DOI:** 10.1002/emmm.201303275

**Published:** 2013-10-08

**Authors:** Kuang-Yung Lee, Moyi Li, Mini Manchanda, Ranjan Batra, Konstantinos Charizanis, Apoorva Mohan, Sonisha A Warren, Christopher M Chamberlain, Dustin Finn, Hannah Hong, Hassan Ashraf, Hideko Kasahara, Laura P W Ranum, Maurice S Swanson

**Affiliations:** 1 Department of Molecular Genetics and Microbiology, Center for NeuroGenetics and the Genetics Institute, University of Florida, College of MedicineGainesville, FL, USA; 2 Department of Neurology, Chang Gung Memorial HospitalKeelung, Taiwan; 3 Department of Physiology and Functional Genomics, University of Florida, College of MedicineGainesville, FL, USA

**Keywords:** Mbnl1, Mbnl2, muscleblind-like, myotonic dystrophy, RNA-mediated disease

## Abstract

Myotonic dystrophy (DM) is a multi-systemic disease that impacts cardiac and skeletal muscle as well as the central nervous system (CNS). DM is unusual because it is an RNA-mediated disorder due to the expression of toxic microsatellite expansion RNAs that alter the activities of RNA processing factors, including the muscleblind-like (MBNL) proteins. While these mutant RNAs inhibit MBNL1 splicing activity in heart and skeletal muscles, *Mbnl1* knockout mice fail to recapitulate the full-range of DM symptoms in these tissues. Here, we generate mouse *Mbnl* compound knockouts to test the hypothesis that Mbnl2 functionally compensates for Mbnl1 loss. Although *Mbnl1*^−/−^; *Mbnl2*^−/−^ double knockouts (DKOs) are embryonic lethal, *Mbnl1*^−/−^; *Mbnl2*^+/−^ mice are viable but develop cardinal features of DM muscle disease including reduced lifespan, heart conduction block, severe myotonia and progressive skeletal muscle weakness. Mbnl2 protein levels are elevated in *Mbnl1*^−/−^ knockouts where Mbnl2 targets Mbnl1-regulated exons. These findings support the hypothesis that compound loss of MBNL function is a critical event in DM pathogenesis and provide novel mouse models to investigate additional pathways disrupted in this RNA-mediated disease.

## INTRODUCTION

Myotonic dystrophy (DM) is an autosomal dominant neuromuscular disease characterized by a multi-systemic phenotype, including skeletal muscle myotonia and progressive weakness/wasting, cardiac arrhythmias, ocular ‘dust-like’ cataracts, insulin insensitivity, hypogammaglobulinemia, hypersomnia and cerebral atrophy (Ranum & Cooper, 2006; Udd & Krahe, 2012). DM is also one of the most variable human hereditary diseases with the age-of-onset ranging from neonates to older adults with nearly all organ systems affected to different degrees.

DM is caused by expansions of either CTG trinucleotide repeats in the 3′ untranslated region (3′ UTR) of the *DMPK* gene on chromosome 19 (DM type 1, DM1) or CCTG repeats in the first intron of the *CNBP* gene on chromosome 3 (DM type 2, DM2) (Ranum & Cooper, 2006; Udd & Krahe, 2012). These non-coding C(C)TG expansion, or C(C)TG^exp^, mutations generate pathogenic C(C)UG^exp^ RNAs that are toxic because they perturb the normal cellular activities of several RNA binding factors, including the muscleblind-like (MBNL), CUGBP1 and ETR3-like (CELF), hnRNP H and STAU1 proteins (Ho et al, 2004; Kanadia et al, 2003; Miller et al, 2000; Paul et al, 2006; Philips et al, 1998; Ravel-Chapuis et al, 2012; Timchenko et al, 1996). The MBNL proteins appear to play a particularly prominent role in DM pathogenesis since each of the three *MBNL* genes (*MBNL1*, *MBNL2*, *MBNL3*) produce multiple isoforms with different, but relatively high, binding affinities for C(C)UG^exp^ RNAs *in vitro* (Kino et al, 2004; Warf & Berglund, 2007; Yuan et al, 2007) and MBNL proteins are sequestered by mutant DM transcripts in nuclear RNA foci *in vivo* (Jiang et al, 2004; Mankodi et al, 2001; Miller et al, 2000). *Mbnl1*^*ΔE3/ΔE3*^, *Mbnl2*^*ΔE2/ΔE2*^ and *Mbnl3*^*ΔE2/ΔE2*^ isoform knockout (KO) mice recapitulate DM-relevant phenotypes with skeletal muscle myotonia, myopathy and particulate cataracts in *Mbnl1*^*ΔE3/ΔE3*^ KOs, increased REM sleep propensity and learning/memory deficits in *Mbnl2*^*ΔE2/ΔE2*^ KOs and an age-associated decline in skeletal muscle regeneration in *Mbnl3*^*ΔE2/ΔE2*^ KO mice (Charizanis et al, 2012; Kanadia et al, 2003; Poulos et al, 2013). *Mbnl1*^*ΔE3/ΔE3*^ and *Mbnl2*^*ΔE2/ΔE2*^ isoform knockout alleles do not express detectable Mbnl1 or Mbnl2 protein, respectively, so for simplicity they will be subsequently referred to as *Mbnl1* and *Mbnl2* KOs or *Mbnl1*^−/−^ and *Mbnl2*^−/−^. Additionally, Mbnl1 overexpression reverses myotonia in transgenic *HSA*^LR^ mice, which express a CUG^exp^ RNA in skeletal muscle, and transgenic MBNL1 overexpression mice are viable with normal muscle structure and function (Chamberlain & Ranum, 2012; Kanadia et al, 2006).

Sequestration and functional inhibition of all three MBNL paralogues depends on C(C)UG^exp^ RNA repeat length and expression level, which are highly variable between tissues. Therefore, we propose that the full range of DM phenotypes cannot be adequately modelled in *Mbnl* single KO mice. Indeed, some prominent DM-relevant manifestations, including skeletal muscle weakness/wasting and cardiac arrhythmias, are not routinely observed in *Mbnl1* and *Mbnl2* single KOs. To test this proposal, we investigated the effects of combined Mbnl1 and Mbnl2 deficiency on cardiac and skeletal muscle structure and function. While *Mbnl1*; *Mbnl2* homozygous double KOs (DKOs) were embryonic lethal, *Mbnl1*^−/−^; *Mbnl2*^+/−^ mice survived until adulthood but developed skeletal and cardiac muscle defects, as well as alternative splicing changes, characteristic of DM. More severe manifestations of DM skeletal muscle disease were observed in *Mbnl1*^−/−^; *Mbnl2*^c/c^; *Myo-Cre*^+/−^ KO mice, with complete absence of Mbnl2 protein expression restricted to muscle fibres. High-throughput sequencing combined with crosslinking/immunoprecipitation (HITS-CLIP), a technique that identifies binding sites for RNA-binding proteins *in vivo*, demonstrated that Mbnl2 upregulation following the loss of *Mbnl1* expression resulted in an increase in Mbnl2 binding to Mbnl1 muscle RNA targets and partial correction of DM-relevant mis-splicing. Our results demonstrate that the major symptoms of cardiac and skeletal muscle disease in DM can be recapitulated in *Mbnl* compound knockout models and that sequestration of both Mbnl1 and Mbnl2 by C(C)UG^exp^ RNAs is an important pathogenic feature of DM.

## RESULTS

### Reduced lifespan in *Mbnl1*; *Mbnl2* compound KO mice

**Figure 1:**
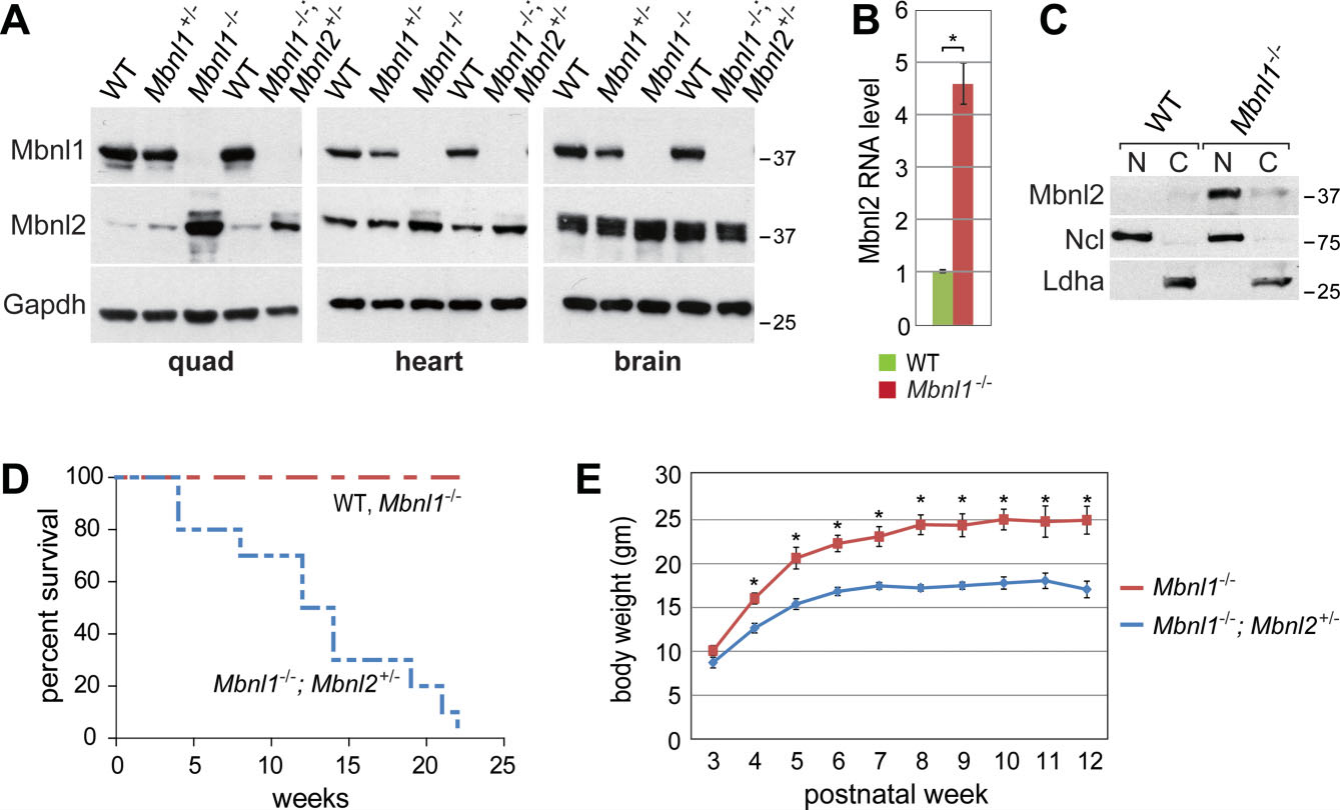
Reduced viability of *Mbnl1*; *Mbnl2* KO mice.

*Mbnl1* KO adults develop skeletal muscle myotonia and DM-associated muscle pathology, including centralized myonuclei and split fibres, but do not display either the hypotonia or adult-onset muscle wasting features of congenital or adult-onset DM, respectively (Kanadia et al, 2003). In contrast, *Mbnl2* KO mice display characteristic manifestations of DM central nervous system (CNS) disease, such as REM sleep propensity disturbance and memory deficits, but do not exhibit muscle abnormalities (Charizanis et al, 2012). Interestingly, we noted that Mbnl2 protein levels increased strikingly in *Mbnl1* KOs, particularly in skeletal muscle (Fig 1A), in agreement with a recent study that reported Mbnl2 protein upregulation following shRNA-mediated knockdown of Mbnl1 in C2C12 myoblasts (Wang et al, 2012). Other factors implicated in DM pathogenesis (Mbnl3, Hnrnph1, Stau1) did not show similar increases in protein level (Supporting Information Fig S1A). The increase in Mbnl2 protein was likely due to a corresponding increase in Mbnl2 mRNA level (Fig 1B). To determine if combined loss of Mbnl1 and Mbnl2 would recapitulate neonatal hypotonia and/or progressive muscle wasting in adults, we attempted to generate *Mbnl1*; *Mbnl2* DKO mice but loss of both proteins resulted in embryonic lethality (Supporting Information Fig S1B). In contrast, *Mbnl1*^−/−^; *Mbnl2*^+/−^, as well as *Mbnl1*^+/−^; *Mbnl2*^−/−^, KOs were viable although the latter line showed reduced embryonic viability so it was not characterized further. While Mbnl2 is low in WT adult muscle, the upregulated Mbnl2 protein in Mbnl1 KO muscle localized primarily to the nuclear compartment (Fig 1C). Elevated nuclear levels of Mbnl2 in the *Mbnl1* KO correlated with increased inclusion of Mbnl2 exons 6 and 8 in skeletal muscle (Supporting Information Fig S2A, B) (Zhang et al, 2013). While the function of Mbnl2 exon 8 is unknown, Mbnl2 exon 6 (54 nt) is homologous to Mbnl1 exon 7 that may encode a nuclear localization sequence (Lin et al, 2006).

A. Immunoblot analysis of *Mbnl1*^+/+^ (WT), *Mbnl1*^+/−^, *Mbnl1*^−/−^ and *Mbnl1*^−/−^; *Mbnl2*^+/−^ KO quadriceps muscle (quad), heart and brain tissues.
B. Upregulation of Mbnl2 RNA in *Mbnl1*^−/−^ KO quadriceps muscle (*n* = 3 per genotype, *p = 0.0008, Student's *t*-test).
C. Immunoblots showing Mbnl2 upregulation in myonuclei of *Mbnl1*^−/−^ KO muscle. Nucleolin (Ncl) and lactate dehydrogenase A (Ldha) were included as nuclear/nucleolar and cytoplasmic markers, respectively.
D. Kaplan–Meier survival analysis of *Mbnl1*^−/−^ KO and WT (red line) *versus Mbnl1*^−/−^; *Mbnl2*^+/−^ (blue) KO mice (*n* = 10 per genotype).
E. Body weight analysis showed *Mbnl1*^−/−^; *Mbnl2*^+/−^ KOs were ∼30% smaller than *Mbnl1*^−/−^ single KOs between 8 and 12 weeks of age (*n* ≥ 7 except for *Mbnl1*^−/−^; *Mbnl2*^+/−^ at 12 weeks of age *n* = 5, *p = 0.00132 (week 4), 0.00047 (week 5), 4.6E-05 (week 6), 0.00013 (week 7), 2.03E-06 (week 8), 2.89E-05 (week 9), 0.00014 (week 10), 0.0002 (week 11), 0.00013 (week 12), Student's *t*-test).

Despite the compensatory increase in Mbnl2 expression in skeletal muscle, lifespan was severely compromised in *Mbnl1*^−/−^; *Mbnl2*^+/−^ KO animals with no mice surviving beyond 23 weeks of age (Fig 1D) and body weight was maintained at ∼30% less than *Mbnl1*^−/−^ (Fig 1E). Since the MBNL loss-of-function model for DM proposes that both MBNL1 and MBNL2 are sequestered to varying levels depending on C(C)UG^exp^ RNA length and expression level, *Mbnl1*^−/−^; *Mbnl2*^+/−^ KO mice were further evaluated for DM-relevant skeletal muscle defects to test the hypothesis that loss of both Mbnl1 and Mbnl2 proteins would increase the severity of skeletal muscle deficits.

### Progressive muscle weakness/wasting and NMJ defects in *Mbnl1*^−/−^; *Mbnl2*^+/−^ KOs

**Figure 2:**
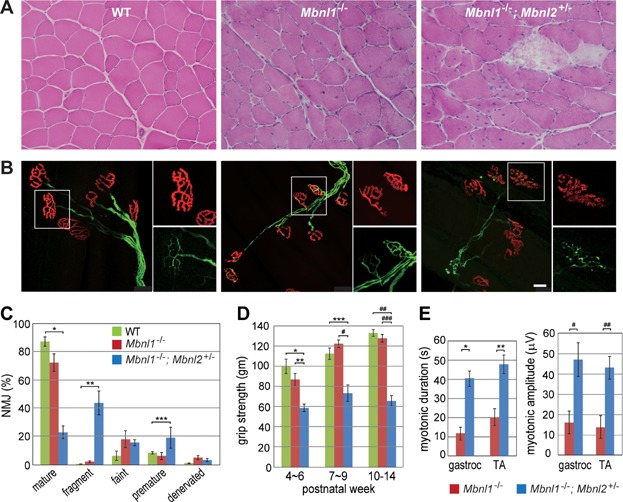
DM-associated muscle wasting/weakness in *Mbnl1*; *Mbnl2* KOs.

Histological hallmarks of DM patient muscle, which include centralized nuclei, fibre size heterogeneity and split fibres, have been previously noted in older (>6 months of age) *Mbnl1* KO quadriceps muscle (Kanadia et al, 2003; Kanadia et al, 2006). While these muscle abnormalities are considerably less severe in younger *Mbnl1* KO mice, more severe pathological changes (increased fibre size variation, atrophic and splitting fibres, increased numbers of central nuclei indicative of muscle degeneration/regeneration) were observed in *Mbnl1*^−/−^; *Mbnl2*^+/−^ muscles at 10–16 weeks of age (Fig 2A). Additionally, young (8–10 weeks of age) *Mbnl1*^−/−^; *Mbnl2*^+/−^ mice developed severe mobility problems and showed impaired rotarod performance (Supporting Information Fig S1C). Abnormalities in diaphragm neuromuscular junctions (NMJs), including changes in end-plate size and shape, have been documented in a poly(CUG) transgenic mouse model for DM1 (Panaite et al, 2008). Similar, but more profound, changes to NMJ structures were observed in *Mbnl1*^−/−^; *Mbnl2*^+/−^ tibialis anterior (TA) muscles but not WT and *Mbnl1*^−/−^ (Fig 2B). Significant loss of mature, and an increase in degenerated (fragmented endplates) and premature, NMJs was observed in *Mbnl1*^−/−^; *Mbnl2*^+/−^ mice (Fig 2B, C). Moreover, grip strength analysis revealed muscle weakness as early as 4 weeks of age, which was half of the WT level by 10 weeks of age (Fig 2D). Myotonia, a characteristic feature of DM muscle, was also dramatically increased in *Mbnl1*^−/−^; *Mbnl2*^+/−^ muscles compared to single *Mbnl1* KO mice with both the amplitude (>4-fold) and duration of discharges increased 2.5 to ∼4-fold (Fig 2E and Supporting Information Fig S1D). Thus, *Mbnl1*^−/−^; *Mbnl2*^+/−^ KOs recapitulate DM-relevant muscle weakness/wasting and myotonia.

A. H&E staining of WT, *Mbnl1*^−/−^ and *Mbnl1*^−/−^; *Mbnl2*^+/−^ TA muscles showing centralized myonuclei, split fibres and myofibre degeneration in young (10–16 weeks of age) *Mbnl1*^−/−^; *Mbnl2*^+/−^ KOs.
B. Mbnl1 and Mbnl2 depletion leads to NMJ defects. WT, *Mbnl1*^−/−^ and *Mbnl1*^−/−^; *Mbnl2*^+/−^ TA muscles were dissected and labelled with α-bungarotoxin (red) and anti-neurofilament H (NFH-1, green). WT NMJs showed mature pretzel-shaped endplates, *Mbnl1*^−/−^ KOs displayed similar features as WT although moderate changes in NMJ structure were noted whereas *Mbnl1*^−/−^; *Mbnl2*^+/−^ KOs showed NMJ fragmentation and a decrease in mature NMJs (scale bar = 25 μm).
C. Percent of NMJs with various morphologies including mature, fragmented (fragment), faint, premature and denervated (*n* = 5 per genotype, *p = 1.9E-07, **p = 1.4E-06, ***p < 0.00827, Student's *t*-test).
D. Age-dependent increase in muscle weakness in *Mbnl1*^−/−^; *Mbnl2*^+/−^ KOs. Grip strength test (forelimb) showed an increase in muscle weakness between 4 and 14 weeks of age (*n* ≥ 6 per genotype, *p = 0.0009, **p = 0.00198, ***p = 0.00161, #*p* = 2.7E-05, ##*p* = 4.6E-06, ###*p* = 1.2E-06, Student's *t*-test).
E. Myotonic duration and amplitude are significantly increased in *Mbnl1*^−/−^; *Mbnl2*^+/−^ KO muscle (*n* = 4, *p = 0.00141, **p = 0.00554, #*p* = 0.02137, ##*p* = 0.0101, Student's *t*-test).

### Combined depletion of Mbnl1 and Mbnl2 exacerbates DM-associated splicing defects

**Figure 3:**
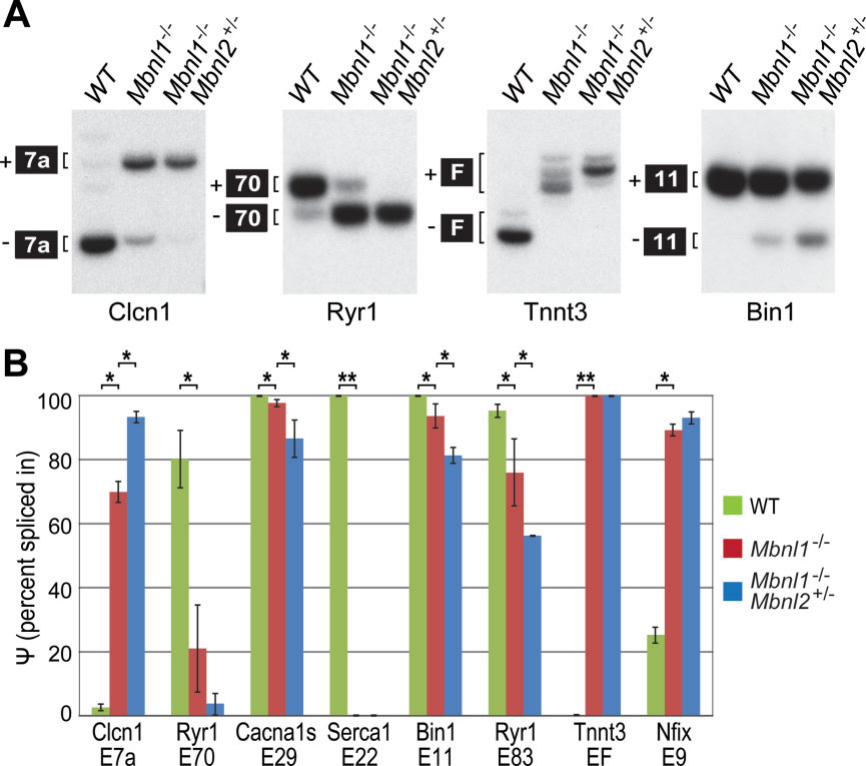
DM-associated splicing deficits following *Mbnl1* and *Mbnl2* depletion.

DM-associated myotonia is caused by mis-regulated splicing of Clcn1 pre-mRNA and depletion of full-length CLCN1 protein from the sarcolemma. Because myotonia was more severe following the depletion of Mbnl2 (Fig 2E), we first compared the Clcn1 splicing pattern in WT, *Mbnl1* KO and *Mbnl1*^−/−^; *Mbnl2*^+/−^ KO skeletal muscle. Depletion of Mbnl2 in the *Mbnl1* KO background led to a nearly complete loss of the adult Clcn1 isoform (−exon 7a) (Fig 3A). Gene profiling studies have highlighted an important role for Mbnl proteins in calcium homeostasis and regulation (Osborne et al, 2009). Several gene transcripts important for calcium regulation, including Ryr1, Serca1 and Cacna1s, were more severely mis-spliced in *Mbnl1*^−/−^; *Mbnl2*^+/−^ KO muscle compared to Mbnl1 KO alone and this degree of mis-splicing was similar to that observed previously in DM1 patient muscle (Kimura et al, 2005; Tang et al, 2012). In addition, Bin1, Tnnt3 and Nfix mis-splicing events, which occur in *Mbnl1* KOs and human DM muscle (Du et al, 2010; Fugier et al, 2011; Kanadia et al, 2003), were also tested. *Mbnl1*^−/−^; *Mbnl2*^+/−^ KO muscle showed either more pronounced splicing changes, or were equivalent to *Mbnl1* KOs (*e.g*. *Serca1*, *Tnnt3*) (Fig 3B and Fig S1E). As reported previously, global effects on alternative splicing regulation are not observed in *Mbnl1* KO mice (Du et al, 2010; Kanadia et al, 2003; Wang et al, 2012) and this was also observed for *Mbnl1*^−/−^; *Mbnl2*^+/−^ KOs (Fig S1F). Collectively, these results demonstrated that combined depletion of Mbnl1 and Mbnl2 recapitulated muscle myotonia, weakness/wasting and the splicing patterns characteristic of severely affected DM1 skeletal muscle (Fugier et al, 2011; Wheeler & Thornton, 2007).

A. RT-PCR analysis of WT, *Mbnl1*^−/−^, *Mbnl1*^−/−^; *Mbnl2*^+/−^ splicing for several pre-mRNA targets that are mis-spliced in DM skeletal muscle. All targets showed enhancement of the fetal isoform splicing pattern in *Mbnl1*^−/−^; *Mbnl2*^+/−^ KOs compared to WT and *Mbnl1*^−/−^ single KOs. The apparent decrease in Clcn1 levels between WT and *Mbnl1*^−/−^ is reproducible (Charizanis et al, 2012) and likely the result of differences in RNA stability. The multiple Tnnt3 +F and −F isoforms result from alternative splicing of exons 4–8 in the absence of *Bsr*B1 digestion of the F exon (Kanadia et al, 2003).
B. Percent spliced-in (*Ψ*) values for eight target pre-mRNAs in WT, *Mbnl1*^−/−^, *Mbnl1*^−/−^; *Mbnl2*^+/−^ quadriceps muscles (*n* = 3 per genotype, *p = 4.4E-06 [Clcn1, WT vs. *Mbnl1*^−/−^], 0.00039 [Clcn1, *Mbnl1*^−/−^ vs. *Mbnl1*^−/−^; *Mbnl2*^+/−^], 0.00323 [Ryr1 E70, WT vs. *Mbnl1*^−/−^], 0.02506 [Cacna1s, WT vs. *Mbnl1*^−/−^], 0.0318 [Cacna1s, *Mbnl1*^−/−^ vs. *Mbnl1*^−/−^; *Mbnl2*^+/−^], 0.04219 [Bin1, WT vs. *Mbnl1*^−/−^], 0.00891 [Bin1, *Mbnl1*^−/−^ vs. *Mbnl1*^−/−^; *Mbnl2*^+/−^], 0.01377 [Ryr1 E83, WT vs. *Mbnl1*^−/−^], 0.03117 [Ryr1 E83, *Mbnl1*^−/−^ vs. *Mbnl1*^−/−^; *Mbnl2*^+/−^], 3.7E-06 [Nfix, WT vs. *Mbnl1*^−/−^], Student's *t*-test; ** no *Ψ* variance was observed for Serca1 and Tnnt3 for WT, *Mbnl1*^−/−^ and *Mbnl1*^−/−^; *Mbnl2*^+/−^).

### *Mbnl1*^−/−^; *Mbnl2*^+/−^ KOs model cardiac conduction defects in DM

Heart conduction abnormalities are common in DM1 patients and cardiac-related sudden death is a leading cause of mortality in this disease (Groh et al, 2008; McNally & Sparano, 2011). Endomyocardial biopsies as well as postmortem examination of DM1 hearts have noted interstitial fibrosis, fatty infiltration, cardiomyocyte hypertrophy and focal myocarditis (Pelargonio et al, 2002). Similarly, interstitial myocardial fibrosis was detected in all *Mbnl1*^−/−^; *Mbnl2*^+/−^, but not all *Mbnl1*^−/−^, KO mice (Supporting Information Fig S3A, B). Fibrosis was also observed in enlarged right atria.

**Figure 4:**
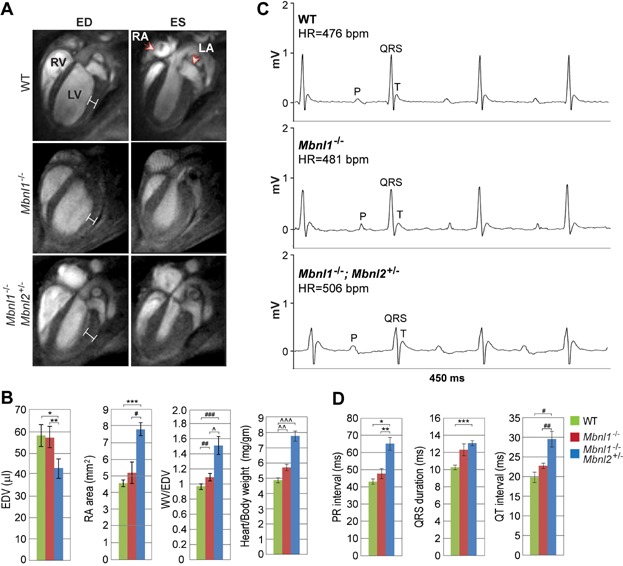
Compound mouse knockout model for DM heart disease.

DM1 patients with cardiac abnormalities generally show various extents of heart chamber dilation and hypertrophy (Hermans et al, 2012; McNally & Sparano, 2011; Pelargonio et al, 2002). MRI analysis of *Mbnl1*^−/−^; *Mbnl2*^+/−^ KOs revealed atrial dilatation and left ventricular hypertrophy (Fig 4A and B). Although compromised myocardial contractility in *Mbnl1*^−/−^; *Mbnl2*^+/−^ KOs was anticipated, the ejection fraction was not significantly different from WT (Supporting Information Fig S3C). *Mbnl1*^−/−^; *Mbnl2*^+/−^ KO hearts were enlarged and heart/body weight (HW/BW) ratios increased ∼60% in *Mbnl1*^−/−^; *Mbnl2*^+/−^ KOs *versus* WT (Fig 4B).

A. MRI cardiac images revealed right atrial (RA) dilatation and thickening of the posterior left ventricular wall in *Mbnl1*^−/−^; *Mbnl2*^+/−^ KOs compared to WT mice while *Mbnl1*^−/−^ mice displayed similar, but less severe, morphological changes.
B. End diastolic volume (EDV), right atrial (RA) area, the ratio of left ventricular wall volume (WV) to end diastolic volume (WV/EDV) and heart/body weight ratio are shown (*n* = 5 per genotype, *p = 0.0043, **p = 0.0139, ***p = 0.001, #*p* = 0.0006, ##*p* = 0.0075, ###*p* = 0.0042, ^*p* = 0.0054, ^^*p* = 0.0035, ^^^*p* = 3.9E-08, Student's *t*-test).
C. Electrocardiography (ECG) evidence for first-degree AV block. Increases in PR interval and QRS broadening were noted compared to WT mice.
D. Significant ECG changes in PR interval, QRS duration and QT interval in WT, *Mbnl1*^−/−^ and *Mbnl1*^−/−^; *Mbnl2*^+/−^ KO hearts are shown (*n* = 5 per genotype, *p = 0.0006, **p = 0.0064, ***p = 0.0001, #*p* = 0.0035, ##*p* = 0.0117, Student's *t*-test).

The majority of DM1 patients are abnormal as assessed by electrocardiography (ECG) with prolonged PR interval (20–40% of patients) and QRS widening (5–25%) (McNally & Sparano, 2011). Indeed, a large-scale clinical study revealed a direct correlation between CTG repeat length, arrhythmias, PR prolongation and QRS widening (Groh et al, 2002). ECG analysis of *Mbnl1*^−/−^; *Mbnl2*^+/−^ KOs showed PR interval prolongation, or an ∼51% increase compared to WT, indicating a first degree AV block in these mutants (Fig 4C and D and Supporting Information Fig S3D). Both QRS duration and QT interval were also significantly increased.

**Figure 5:**
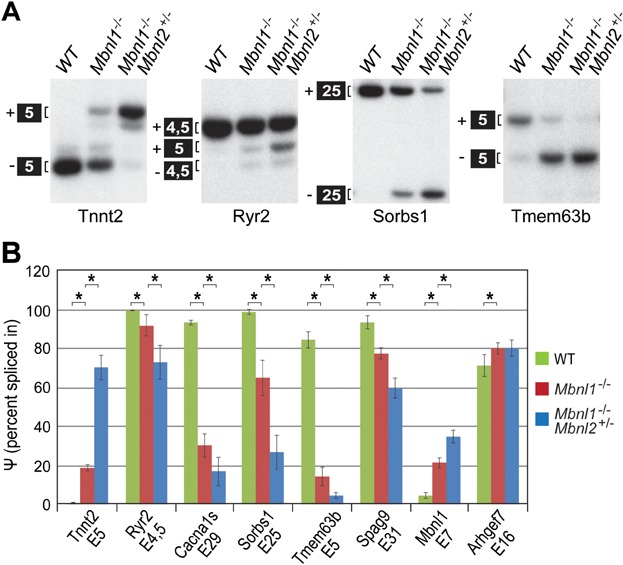
Splicing alterations of cardiac RNA targets in *Mbnl1*^−/−^; *Mbnl2*^+/−^ KOs.

Since depletion of Mbnl2 in the absence of Mbnl1 resulted in structural and functional alterations to the heart, DM-associated mis-splicing events for Mbnl1 heart splicing targets were evaluated by RT-PCR. For all targets examined, *Mbnl1*^−/−^; *Mbnl2*^+/−^ KOs showed a dramatic shift towards fetal isoforms (Fig 5A and Supporting Information Fig S3E). For example, the fetal isoform of Tnnt2 includes exon 5 and splicing of this exon increased from ∼0% in WT to >70% in *Mbnl1*^−/−^; *Mbnl2*^+/−^ KOs. Enhanced mis-splicing also occurred for Ryr2, which regulates calcium release from the sarcoplasmic reticulum and *RYR2* mutations cause catecholaminergic polymorphic ventricular tachycardia (Venetucci et al, 2012), and Cacna1s, which was recently shown to be mis-spliced in cardiac and skeletal muscle leading to aberrant gating of this Ca(V)1.1 calcium channel (Tang et al, 2012). Both of these Mbnl1 splicing targets, as well as additional targets (Sorbs1, Tmem63b, Spag9, Mbnl1), showed augmentation of mis-splicing changes in *Mbnl1*^−/−^; *Mbnl2*^+/−^ KOs compared to *Mbnl1* KO alone with the single exception of Arhgef7 (Fig 5B).

A. Increased mis-splicing of the Mbnl1 pre-mRNA targets Tnnt2, Ryr2, Sorbs1 and Tmem63b occurs in *Mbnl1*^−/−^; *Mbnl2*^+/−^ compound KOs compared to WT or *Mbnl1* single KO mice.
B. Percent spliced-in (*Ψ*) values for eight target pre-mRNAs in WT, *Mbnl1*^−/−^, *Mbnl1*^−/−^; *Mbnl2*^+/−^ hearts (*n* = 3 per genotype, *p = 5.3E-05 [Tnnt2, WT vs. *Mbnl1*^−/−^], 0.00023 [Tnnt2, *Mbnl1*^−/−^ vs. *Mbnl1*^−/−^; *Mbnl2*^+/−^], 0.03988 [Ryr2, WT vs. *Mbnl1*^−/−^], 0.028 [Ryr2, *Mbnl1*^−/−^ vs. *Mbnl1*^−/−^; *Mbnl2*^+/−^], 5.5E-05 [Cacna1s, WT vs. *Mbnl1*^−/−^], 0.02365 [Cacna1s, *Mbnl1*^−/−^ vs. *Mbnl1*^−/−^; *Mbnl2*^+/−^], 0.0035 [Sorbs1, WT vs. *Mbnl1*^−/−^], 0.0074 [Sorbs1, *Mbnl1*^−/−^ vs. *Mbnl1*^−/−^; *Mbnl2*^+/−^], 4.8E-05 [Tmem63b, WT vs. *Mbnl1*^−/−^], 0.0296 [Tmem63b, *Mbnl1*^−/−^ vs. *Mbnl1*^−/−^; *Mbnl2*^+/−^], 0.0035 [Spag9, WT vs. *Mbnl1*^−/−^], 0.0076 [Spag9, *Mbnl1*^−/−^ vs. *Mbnl1*^−/−^; *Mbnl2*^+/−^], 0.00076 [Mbnl1, WT vs. *Mbnl1*^−/−^], 0.00692 [Mbnl1, *Mbnl1*^−/−^ vs. *Mbnl1*^−/−^; *Mbnl2*^+/−^], 0.048 [Arhgef7, WT vs. *Mbnl1*^−/−^], Student's *t*-test). Targets are implicated in calcium homeostasis and contractility (Tnnt2, Ryr2, Cacna1s), insulin (Sorbs1), VEGF (Tmem63b) and JNK (Spag9) signalling pathways.

### *Mbnl* conditional compound loss of function model for DM

**Figure 6:**
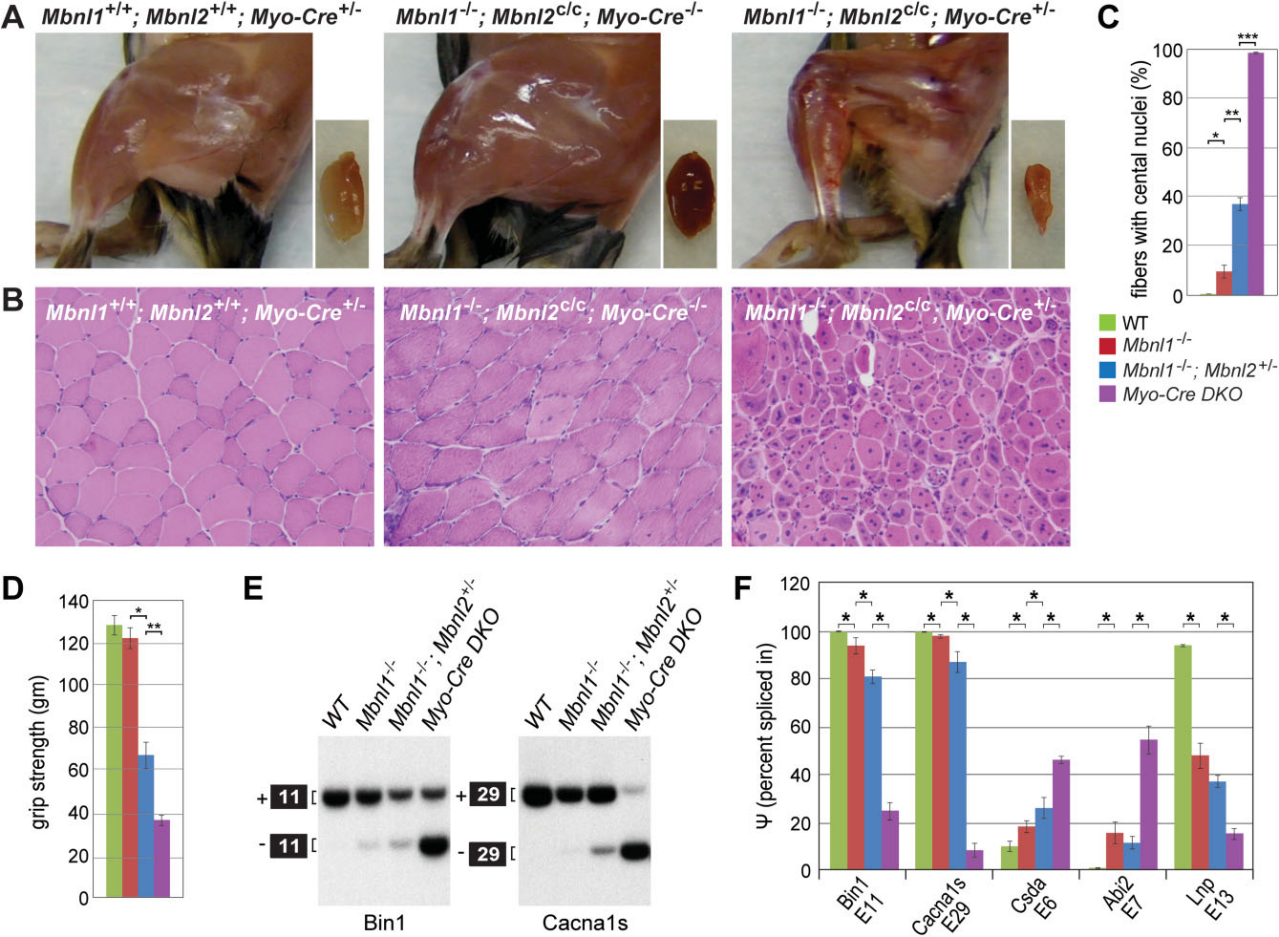
Enhanced muscle pathology in *Mbnl1*; *Mbnl2* muscle-specific KO mice.

Although *Mbnl1*^−/−^; *Mbnl2*^+/−^ KOs displayed decreased lifespan and DM-associated changes in skeletal and cardiac muscle structure and function, Mbnl2 protein levels remained elevated over WT (Fig 1A). This result suggested that Mbnl2 levels increased as a compensatory response to Mbnl1 loss to maintain at least some expression of adult-specific isoforms. However, MBNL1 and MBNL2 have also been recently implicated as negative regulators of pluripotency since they repress ES cell-specific alternative splicing of key regulatory factors, including FOXP1 (Han et al, 2013). Since Mbnl2 protein expression normally declines during muscle development (Charizanis et al, 2012), elevated Mbnl2 levels in *Mbnl1*^−/−^ and *Mbnl1*^−/−^; *Mbnl2*^+/−^ KOs may not be due to compensatory upregulation but instead might reflect an early developmental deficiency and a more immature tissue phenotype in these mutant adults. This finding prompted us to examine the effect of muscle-specific elimination of Mbnl2 protein expression in the *Mbnl1* KO background later in development using a Cre-mediated conditional knockout strategy. Similar to *Mbnl1*^−/−^; *Mbnl2*^+/−^ KOs, *Mbnl1*^−/−^; *Mbnl2*^c/c^; *Myo-Cre*^+/−^ KO mice were small at birth but developed kyphosis and severe motor deficits beginning at 12 weeks of age. In agreement with the hypothesis that complete loss of Mbnl1 and Mbnl2 protein expression would exacerbate muscle pathology and splicing defects, *Mbnl1*^−/−^; *Mbnl2*^c/c^; *Myo-Cre*^+/−^ KO mice showed a profound deficiency of adult skeletal muscle (Fig 6A), severe muscle pathology (small myofibers, fibre size heterogeneity, centralized nuclei in nearly all myofibers) (Fig 6B, C) and reduced grip strength (Fig 6D). Moreover, the alternative splicing of specific pre-mRNAs, including Bin1 (Fugier et al, 2011) and Cacna1s (Tang et al, 2012), previously associated with defects in skeletal muscle excitation-contraction coupling and muscle weakness/wasting shifted to more CDM and DM1-like splicing patterns with only ∼25% of Bin1 (+exon 11) and ∼10% Cacna1s (+exon 29) adult isoforms expressed in *Mbnl1*^−/−^; *Mbnl2*^c/c^; *Myo-Cre*^+/−^ KO muscle (Fig 6E, F). These results provided additional support for the MBNL compound loss-of-function model for DM and suggested that Mbnl conditional KOs will be valuable tools to study DM disease pathways.

A. Images of left hindlimb (left large panels), and dissected quadripceps muscles (small right panels), of WT (*Mbnl1*^+/+^; *Mbnl2*^+/+^; *Myo-Cre*^+/−^), *Mbnl1* KO (*Mbnl1*^−/−^; *Mbnl2*^c/c^; *Myo-Cre*^−/−^) and *Mbnl1*^−/−^; *Mbnl2*^c/c^; *Myo-Cre*^+/−^ DKOs.
B. H&E staining of transverse muscle sections in tibialis anterior (TA) muscles of WT (*Mbnl1*^+/+^; *Mbnl2*^+/+^; *Myo-Cre*^+/−^), *Mbnl1* KO (*Mbnl1*^−/−^; *Mbnl2*^c/c^; *Myo-Cre*^−/−^) and *Mbnl1*^−/−^; *Mbnl2*^c/c^; *Myo-Cre*^+/−^ DKOs.
C. Increased centralized myonuclei in *Mbnl1*^−/−^; *Mbnl2*^c/c^; *Myo-Cre*^+/−^ DKOs (purple) *versus* WT (green, *Mbnl1*^+/+^; *Mbnl2*^+/+^; *Myo-Cre*^+/−^), *Mbnl1*^−/−^ KO (red, *Mbnl1*^−/−^; *Mbnl2*^c/c^; *Myo-Cre*^−/−^) and *Mbnl1*^−/−^; *Mbnl2*^+/−^ (blue) mice (*n* = 2 per genotype, *p = 0.00208, **p = 9.3E-06, ***p = 2.7E-10, Student's *t*-test).
D. Decreased grip strength in *Mbnl1*^−/−^; *Mbnl2*^c/c^; *Myo-Cre*^+/−^ DKOs (purple) *versus* WT (green), *Mbnl1* KO (red) and *Mbnl1*^−/−^; *Mbnl2*^+/−^ (blue) mice (15 weeks of age, *n* = 11 for WT and *Mbnl1*^−/−^, 5 for *Mbnl1*^−/−^; *Mbnl2*^+/−^ and 2 for *Myo-Cre*^+/−^ DKO; *p = 7.7E-06, **p = 0.03337, Student's *t*-test).
E. RT-PCR analysis showing profound increases in Bin1 exon 11 and Cacna1s exon 29 skipping when the expression of both Mbnl1 and Mbnl2 is eliminated in *Mbnl1*^−/−^; *Mbnl2*^c/c^; *Myo-Cre*^+/−^ DKO (Myo-cre DKO) skeletal muscle.
F. Percent spliced-in (*Ψ*) values for five target pre-mRNAs in WT (green), *Mbnl1*^−/−^ (red), *Mbnl1*^−/−^; *Mbnl2*^*+/−*^ (blue) and *Mbnl1*^−/−^; *Mbnl2*^c/c^; *Myo-Cre*^+/−^ DKO (*Myo-Cre* DKO) quadriceps muscles (*n* ≥ 2, *p = 0.0069 [Bin1, WT vs. *Mbnl1*^−/−^], 0.00047 [Bin1, *Mbnl1*^−/−^ vs. *Mbnl1*^−/−^; *Mbnl2*^+/−^], 8.7E-06 [Bin1, *Mbnl1*^−/−^; *Mbnl2*^+/−^ vs. *Myo-Cre*^+/−^ DKO], 0.00078 [Cacna1s, WT vs. *Mbnl1*^−/−^], 0.00158 [Cacna1s, *Mbnl1*^−/−^ vs. *Mbnl1*^−/−^; *Mbnl2*^+/−^], 2.1E-06 [Cacna1s, *Mbnl1*^−/−^; *Mbnl2*^+/−^ vs. *Myo-Cre*^+/−^ DKO], 0.00055 [Csda, WT vs. *Mbnl1*^−/−^], 0.00809 [Csda, *Mbnl1*^−/−^ vs. *Mbnl1*^−/−^; *Mbnl2*^+/−^], 0.00108 [Csda, *Mbnl1*^−/−^; *Mbnl2*^+/−^ vs. *Myo-Cre*^+/−^ DKO], 7.2E-05 [Abi2, WT vs. *Mbnl1*^−/−^], 0.00876 [Abi2, *Mbnl1*^−/−^; *Mbnl2*^+/−^ vs. *Myo-Cre*^+/−^ DKO], 0.006 [Lnp, WT vs. *Mbnl1*^−/−^], 0.00825 [Lnp, *Mbnl1*^−/−^; *Mbnl2*^+/−^ vs. *Myo-Cre*^+/−^ DKO], Student's *t*-test).

### Splicing compensation between Mbnl proteins *in vivo*

**Figure 7:**
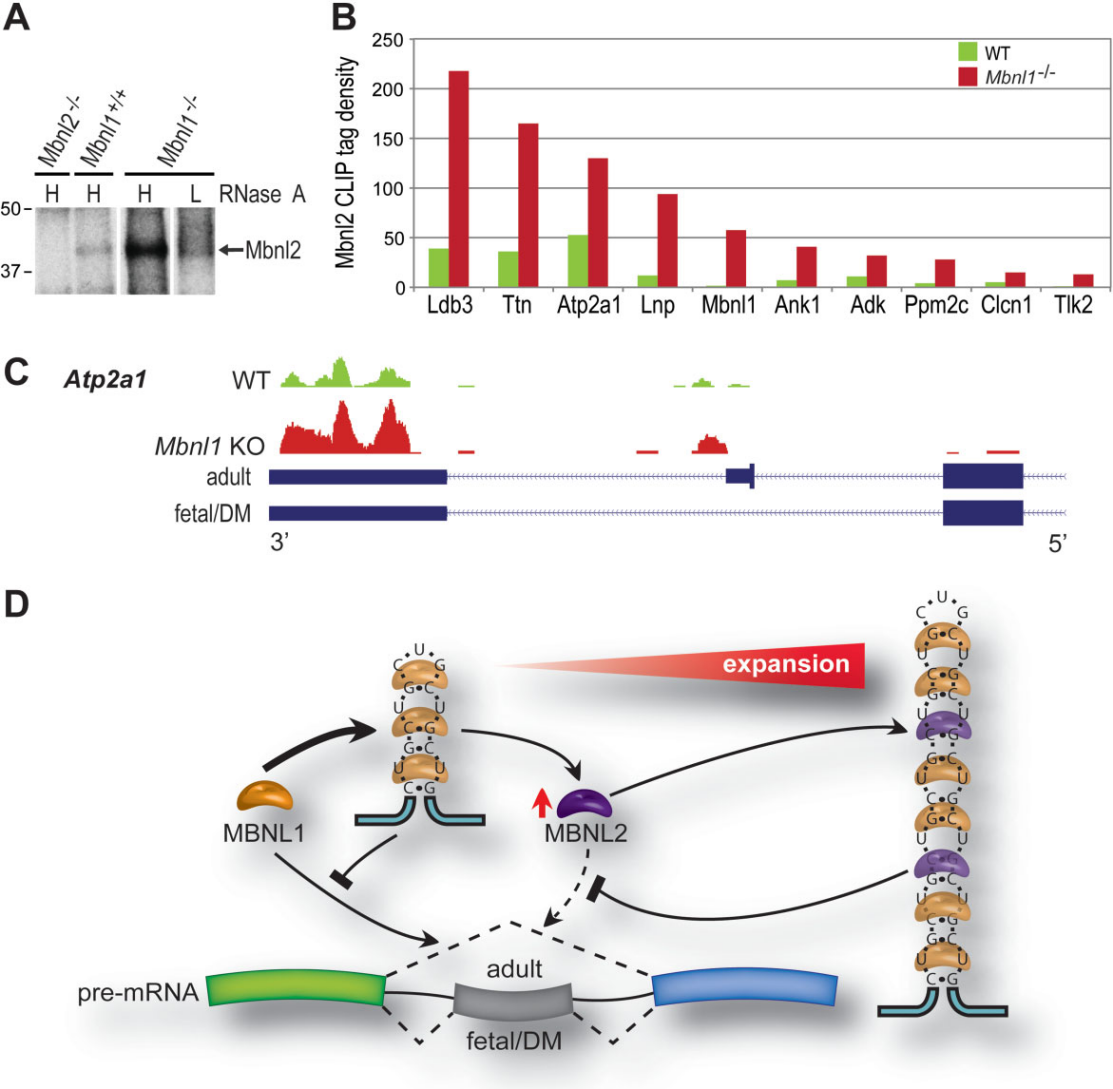
HITS-CLIP analysis in *Mbnl1* KO mice.

Loss of Mbnl1 in *Mbnl1* KO mice led to Mbnl2 protein upregulation in the nucleus while *Mbnl1*^−/−^; *Mbnl2*^+/−^ KOs showed an enhancement of the skeletal and cardiac muscle phenotypes characteristic of DM. These results suggested that Mbnl2 functionally compensated for Mbnl1 loss by targeting Mbnl1-regulated splicing events. To test this possibility, Mbnl protein-RNA interactions were assessed *in vivo* using HITS-CLIP. *Mbnl1*^+/+^, *Mbnl1*^−/−^ and *Mbnl2*^−/−^ quadriceps muscles were dissected, immediately powdered in liquid nitrogen and irradiated with UV-light to form covalently crosslinked protein-RNA complexes. Following digestion with RNase A to generate short RNA tags and RNA end-labelling with ^32^P-ATP, Mbnl2-RNA complexes were immunopurified and resolved by SDS-PAGE (Fig 7A). At high RNase, Mbnl2 migrated at ∼40 kDa and this band was absent in *Mbnl2* KO muscle but increased significantly in *Mbnl1* KO quadriceps in agreement with the earlier immunoblot analysis (Fig 1A). Sequencing of RNA tags bound directly to Mbnl2 in muscle demonstrated a 2- 9-fold increase in Mbnl2 binding to previously documented Mbnl1 skeletal muscle RNA targets (Fig 7B) (Du et al, 2010). Of 667 significantly misspliced targets, 169 (∼25%) showed increased Mbnl2 binding in *Mbnl1* KO mice as compared to WT. Gene ontology (GO) analysis of these targets revealed enrichment for phosphoproteins, alternative splicing factors, ion binding factors involved in calcium homeostasis, ion channel regulation and cytoskeletal organization. Mapping analysis of these targets showed elevated binding of Mbnl2 in the vicinity of alternatively spliced exons in *Mbnl1* KO mice compared to WT (Fig 7C). Therefore, Mbnl2 compensates for the loss of Mbnl1 splicing activity by enhanced binding to Mbnl1-regulated splicing targets resulting in partial retention of the adult splicing pattern.

A. Autoradiography showing increased Mbnl2 protein crosslinking to P^32^-labelled RNA tags from *Mbnl1* KO quadriceps muscle *versus* WT. RNA tags were generated using either high (H) or low (L) RNase A concentrations. *Mbnl2* KO (*Mbnl2*^−/−^) muscle was included as a crosslinking control.
B. Increased Mbnl2 CLIP tag density following Mbnl1 loss in *Mbnl1*^−/−^ KO muscle for 10 *Mbnl1* splicing targets (*n* = 3 for each genotype).
C. UCSC Genome Browser snapshot of Atp2a1 with wiggle plots of Mbnl2 CLIP tags showing increased Mbnl2 binding on Mbnl1-regulated targets in *Mbnl1*^−/−^ *versus* WT muscle.
D. MBNL protein titration model for DM. In muscle, MBNL1 is the primary MBNL protein that promotes adult splicing patterns (dotted lines depict adult skipping and fetal/DM inclusion of a fetal exon). Early in disease progression, relatively short C(C)UG^exp^ RNAs sequester MBNL1 which, in turn, leads to MBNL2 upregulation (red arrow) and partial mitigation (dotted arrow) of the spliceopathy. However, as C(C)UG^exp^ lengths increase, the splicing activities of both MBNL1 and MBNL2 are blocked (line with bar) leading to loss of adult splicing and more severe disease manifestations.

## DISCUSSION

Defects in DNA replication, repair and recombination cause microsatellite expansions in DM1 and DM2 (Lopez Castel et al, 2010; Mirkin, 2007). Both the *DMPK* and *CNBP* genes are broadly expressed and microsatellite C(C)TG expansions are dynamic, tend to increase with age and show a high degree of somatic mosaicism (Higham et al, 2012; Morales et al, 2012). Because different tissues, and even individual cells within tissues, contain different repeat lengths and varying degrees of pathology, the development of animal models that faithfully recapitulate the consequences of these microsatellite expansions has been exceptionally challenging.

Transgenic poly(CUG) mouse models have provided key insights into the DM pathogenic mechanism (Sicot & Gomes-Pereira, 2013). For example, *HSA*^LR^ transgenic mice, which express an expanded (CUG)_∼220_ repeat only in skeletal muscles, develop multiple DM-like muscle abnormalities, including myotonia, fibre size variability, split fibres and centralized myonuclei although they fail to display DM-like severe muscle weakness and wasting perhaps due to the relatively short CUG^exp^ length (Mankodi et al, 2000). Interestingly, a recent study implicated mis-splicing of exon 29 in CACNA1S, a calcium channel that regulates excitation-contraction coupling, in DM muscle weakness/wasting but poly(CUG) expression in *HSA*^LR^ muscle only moderately affects this splicing event (Tang et al, 2012). The multi-systemic features of DM are reproduced in the DM300 (CTG_300-600_) and DMSXL (CTG_1000-1800_) transgenic models although disease-relevant phenotypes and RNA mis-splicing are relatively mild (Sicot & Gomes-Pereira, 2013). In contrast, tissue-specific and Cre-inducible EpA960 transgenic mice, which express the DMPK 3′ UTR with an interrupted CTG_960_ repeat at relatively high levels, display severe cardiac arrhythmias, muscle wasting and splicing defects (Orengo et al, 2008; Wang et al, 2007).

Since transcription of mutant *DMPK* and *CNBP* genes results in the production of pathogenic C(C)UG repeat RNAs that dysregulate gene expression by altering the activities of several RNA-binding proteins, another approach has been to generate Celf1 gain-of-function (Koshelev et al, 2010; Ward et al, 2010) and Mbnl loss-of function models (Charizanis et al, 2012; Kanadia et al, 2003; Poulos et al, 2013). Because MBNL proteins are the only factors which have been shown to be sequestered by C(C)UG RNAs, the primary objective of this study was to determine if compound depletion of Mbnl function recapitulates disease-relevant skeletal and cardiac muscle manifestations. Previously, we demonstrated that *Mbnl2* muscle expression decreases during postnatal development and *Mbnl2* KO mice fail to exhibit the major muscle features of DM (Charizanis et al, 2012). Here, we show that loss of *Mbnl1* gene expression leads to upregulation of *Mbnl2*, which subsequently targets those splicing events that are normally regulated primarily by Mbnl1. This is reminiscent of other alternative splicing factors, including the Rbfox family in which Rbfox2 expression is upregulated in the absence of Rbfox1 and vice versa (Gehman et al, 2012; Gehman et al, 2011). This compensatory Mbnl2 upregulation may be essential for *Mbnl1* KO viability since *Mbnl1*; *Mbnl2* DKOs were embryonic lethal. Additionally, *Mbnl1*^−/−^; *Mbnl2*^+/−^ KOs displayed muscle weakness and progressive atrophy, two of the common symptoms observed in DM1 patients.

DM1 patients are prone to sudden death due to cardiac arrhythmias (Groh et al, 2008; Motta et al, 1979). *Mbnl1*^−/−^; *Mbnl2*^+/−^ KOs also developed these cardiac symptoms and DM-associated mis-splicing events increased for a number of key target RNAs, including Tnnt2, Ryr2 and Cacna1s in the heart and Clcn1, Ryr1 and Tnnt3 in skeletal muscle. Interestingly, *Mbnl1*^−/−^ KO mice showed a more modest increase of Mbnl2 in the brain (Fig 1A) and previous studies have reported that mis-splicing of several brain RNA targets in this knockout model was significantly less than observed in human DM1 brain (Suenaga et al, 2012) or in *Mbnl2*^−/−^ KO mice (Charizanis et al, 2012). Although this study focused on the role of MBNL proteins in the regulation of alternative splicing in skeletal muscle and heart, the development of additional *Mbnl1*; *Mbnl2* conditional KO models should allow assessment of the relative contributions of each *Mbnl* gene to CNS function. It is also interesting to note that similar to DMSXL homozygotes, which express large CUG^exp^ RNAs, *Mbnl2*^−/−^, *Mbnl1*^−/−^; *Mbnl2*^+/−^ and *Mbnl1*^−/−^; *Mbnl2*^c/c^; *Myo-Cre*^+/−^ KO mice are small at birth whereas *Mbnl1*^−/−^ KOs are not. Therefore, Mbnl2 expression in the embryonic muscle compartment appears to be a critical factor in the normal development of body size. Finally, MBNL1 has been implicated in altered miRNA biogenesis (Rau et al, 2011) and RNA localization (Wang et al, 2012) in DM so it will be important to examine the effects of compound Mbnl loss on these regulatory pathways.

MBNL1 is the most abundant MBNL protein in adult skeletal muscle and plays the predominant role in alternative splicing regulation in muscle while MBNL2 muscle levels decrease during postnatal development (Charizanis et al, 2012; Holt et al, 2009; Wang et al, 2012). These observations suggest a model in which sequestration of MBNL1 by relatively short C(C)UG^exp^ RNAs in early-stage muscle disease results in MBNL2 upregulation and partial restoration of the normal adult splicing program (Fig 7D). However, progressive increases in C(C)UG^exp^ lengths overwhelms this compensatory mechanism leading to impairment of MBNL-driven adult splicing patterns and more severe pathological consequences. In summary, our results provide important new experimental support for the MBNL loss-of-function model for DM and introduce new mouse models to examine the tissue-specific consequences of MBNL loss.

## MATERIALS AND METHODS

### Mouse *Mbnl* KO models

Detailed information on the generation of *Mbnl1* and *Mbnl2* KO mice has been published (Charizanis et al, 2012; Kanadia et al, 2003). Compound KOs were generated by crossing *Mbnl1*^*+/ΔE3*^ and *Mbnl2*^+/*ΔE2*^ heterozygotes (C57BL/129 background) due to fertility issues with homozygous *Mbnl1* and *Mbnl2* KOs. Muscle-specific *Mbnl2* KO mice were produced using *Myo-Cre* transgenic mice (gift of E. Olson, University of Texas Southwestern Medical Center) (Li et al, 2005). *Mbnl1*^*ΔE3*/*ΔE3*^; *Mbnl2*^c/c^; *Myo-Cre*^+/−^ mice were generated by crossing *Mbnl1*^+/*ΔE3*^; *Mbnl2*^c/c^; *Myo-Cre*^+/−^ males with *Mbnl1*^+/*ΔE3*^; *Mbnl2*^c/c^; *Myo-Cre*^−/−^ females. Controls included *Mbnl1*^+/+^; *Mbnl2*^+/+^; *Myo-Cre*^+/−^ (WT control) and *Mbnl1*^*ΔE3*/*ΔE3*^; *Mbnl2*^c/c^; *Myo-Cre*^−/−^ (*Mbnl1* KO control) mice. All animal procedures were reviewed and approved by the University of Florida IACUC.

### Immunoblotting and subcellular fractionation

Dissected tissues from WT (*Mbnl1*^+/+^), *Mbnl1*^+/*ΔE3*^ (*Mbnl1*^+/−^), *Mbnl1*^*ΔE3/ΔE3*^; *Mbnl2*^+/*ΔE2*^ (*Mbnl1*^−/−^; *Mbnl2*^+/−^) (4–5 months of age) skeletal muscles (quadriceps) and hearts were homogenized (Polytron, Kinematica) in lysis buffer [20 mM HEPES-KOH, pH 8.0, 100 mM KCl, 0.1% Igepal CA-630 (Sigma), 0.5 mM phenylmethysulphonyl fluoride, 5 μg/ml pepstain A, 1 μg/ml chymostatin, 1 mM ɛ-aminocaproic acid, 1 mM *p*-aminobenzamidine, 1 μg/ml leupeptin, 2 μg/ml aprotinin] on ice, followed by sonication and centrifugation (16,100*g*, 15 min, 4°C) as described previously (Charizanis et al, 2012). Proteins were detected by immunoblotting (50 μg lysate/lane) using rabbit polyclonal antibody (rpAb) anti-Mbnl1 A2764 (gift of C. Thornton, University of Rochester), mouse monoclonal (mAb) anti-Mbnl2 3B4 (Santa Cruz Biotechnology), anti-Gapdh mAb 6C5 (Abcam), rpAb anti-nucleolin (Abcam), rpAb anti-Ldha (Cell Signaling Technology), rpAb anti-Hnrnph1 (Novus), rpAb anti-Stau1 (Abcam), rpAb anti-Mbnl3 (Poulos et al, 2013) and HRP-conjugated anti-mouse, or anti-rabbit, secondary antibody followed by ECL (GE Healthcare). Subcellular fractionations were performed using NE-PER Nuclear and Cytoplasmic Extraction Reagents according to the manufacturer's protocol (Thermo Scientific) using *Mbnl1*^+/+^ and *Mbnl1*^−/−^ quadriceps muscle.

### Grip strength and electromyography (EMG)

Mice (*n* = 6 for each genotype and time point) were assessed for forelimb grip strength using a grip strength meter (Columbia Instruments). EMG was performed on *Mbnl1*^−/−^, *Mbnl1*^−/−^; *Mbnl2*^+/−^ (2–3 months of age, *n* = 4 per genotype) skeletal muscles (gastrocnemius, TA) as described previously (Chamberlain & Ranum, 2012; Kanadia et al, 2006). Mice were placed on a temperature-controlled heating pad and 30G concentric needle electrodes (CareFusion Teca Elite, *n* = 4–5 insertions/muscle) were used with the TECASynergy EMG system (VIASYS Healthcare). Myotonic amplitudes were measured 8 s post-insertion.

### Muscle histology, NMJ analysis and immunohistochemistry

Frozen skeletal muscle transverse sections (10 μm) of TA muscle were stained with hematoxylin and eosin (H&E) to determine the extent of DM-relevant muscle pathology. For cardiac analysis, hearts were fixed with 4% paraformaldehyde and longitudinal sections (5 μm) were stained by H&E or Masson's trichrome.

For NMJ structural analysis, *Mbnl1*^+/+^, *Mbnl1*^−/−^ and *Mbnl1*^−/−^; *Mbnl2*^+/−^ (4–5 months of age) TA muscles were dissected and fixed in 2% paraformaldehyde at 4°C overnight and then the myofibers were teased off into 5–10 thinner muscle bundles. For histological analysis, acetylcholine receptors were stained with 1 μg/ml α-bungarotoxin conjugated with Alexa Fluor 594 (Invitrogen). To detect axons and synapses, muscle fibres were incubated with chicken polyclonal antibodies against neurofilament H (NF-H, EnCor Biotechnology, 1:2000 dilution) and followed by secondary antibody conjugated with Alexa Fluor 488 (1:500 dilution) (Invitrogen). For NMJ quantification, >100 NMJs per genotype were counted and categorized into four groups previously described for NMJ morphological development including mature (pretzel-shaped endplates with branched innervating axons), fragment (endplate fragmentation), faint (endplate with low α-bungarotoxin staining), premature (pre-maturation stages including plaque, perforated plaque, c-shape, branched) and denervated (no endplate synapses) (Kummer et al, 2004; Sahashi et al, 2012; Valdez et al, 2010).

### RNA splicing and qRT-PCR

Mouse WT, *Mbnl1*^−/−^, *Mbnl1*^−/−^; *Mbnl2*^+/−^ (4–5 months of age) skeletal muscle (quadriceps) and heart RNAs, as well as *Mbnl1*^+/+^; *Mbnl2*^+/+^; *Myo-Cre*^+/−^ and *Mbnl1*^−/−^; *Mbnl2*^c/c^; *Myo-Cre*^+/−^ skeletal muscle RNAs, were isolated using TRI Reagent (Sigma–Aldrich) and cDNAs were prepared using 5 µg of RNA and SuperScript III RT (Invitrogen) according to manufacturer's protocol. For analysis of alternative splicing, specific PCR primers were used (Supporting Information Table S1) and the products were labelled with [α^32^P]-dCTP (2.5 μCi/reaction, PerkinElmer Life Sciences) followed by autoradiography and phosphorimaging to calculate the *Ψ* value (percent spliced in) as described previously (Suenaga et al, 2012).

Quantitative RT-PCR to determine Mbnl2 RNA levels was performed using RNAs isolated from *Mbnl1*^+/+^ and *Mbnl1*^−/−^ quadriceps muscle and the MyiQ real-time PCR detection system (Biorad). First-strand cDNA was prepared as described above and Mbnl2 transcripts containing exon 2 were amplified using 2XiQ SYBR Green Supermix (Biorad) and 250 nM of the MSS4595 and MSS4596 primers (Supporting Information Table S1). Gapdh amplicons, generated using MSS4178 and MSS4179, were used for normalization. Real-time PCR reaction conditions were 95.0°C for 10 min, 45 cycles of 95.0°C for 15 s, 55.0°C for 30 s and 72.0°C for 30 s followed by 7 min at 72°C.

### ECG and MRI heart analysis

Detail procedures for ECG acquisition have been described previously (Gehrmann & Berul, 2000; Kasahara et al, 2001). Briefly, mice were anesthetized with 1.5% isoflurane and the ECG was recorded using six surface leads. ECG recordings were acquired using a multichannel amplifier followed by conversion to digital signals for analysis. (MAClab system, AD Instruments). Corrected QT interval was calculated using Mitchell's formula (Mitchell et al, 1998).

ECG-gated Cine Cardiac MRI has also been described previously (Slawson et al, 1998; Warren et al, 2012). Mice were anesthetized with 1.5% isoflurane and a warm air fan (SA Instruments) was used to maintain stable body temperature. MRI images were acquired using a 4.7 Tesla (bore size 33 cm) horizontal scanner (Agilent). This system includes a 12 cm inner diameter active-shield gradient coil and has a 40 G/cm gradient strength and rise-time of 135 µs. A home-made quadrature saddle-shaped transceiver surface coil of 20 × 30 mm^2^ in size was used. Mice were positioned with the heart in the magnet iso-centre and the long- and short-axis orientation of the heart was scouted using a gradient-echo sequence. Shimming and pulse calibration were performed automatically prior to each experiment. Cine-FLASH was used to acquire temporally resolved dynamic short-axis and long-axis images of the heart with the following parameters: TReff = RR-interval, TE = 1.8 ms, flip angle = 30°, field of view, 25 × 25 mm^2^; acquisition matrix, 128 × 128 pixels; slice thickness, 1 mm. The number of frames per cardiac cycle was adapted to the heart rate of each animal to encompass the entire cardiac cycle.

To calculate the left ventricle ejection fraction (LVEF), short axis images from apex to bottom were acquired and endocardial contours were manually traced in end-diastole and in end-systole using ImageJ. The left ventricle cavity volume was calculated as the sum of the cavity areas multiplied by the section thickness (Lalande et al, 2004; Malm et al, 2004). Papillary muscles and trabeculations were, according to the American Society of Echocardiography (ASE) criteria, included in the LV cavity (Malm et al, 2004). LVEF was calculated based on LVEF% = (EDV_LV_ − ESV_LV_)/EDV_LV_ × 100, where EDV_LV_ is the end-diastolic volume of the left ventricle, ESV_LV_ is the end-systolic volume of the left ventricle. Right atrial (RA) area was traced and calculated using the method described by Prakken (Prakken et al, 2011). The extent of LV hypertrophy was indicated by the ratio of left ventricular wall volume (WV) to EDV_LV_.

### Rotarod analysis

Mice (8–10 weeks of age, *n* = 5 per group) were allowed to acclimate in a behavioural facility for 30 min and then placed on an accelerating rotarod (Accuscan Instruments) rotating at 4 RPM which was gradually increased to 40 RPM over 5 min and then continued at 40 RPM for an additional 5 min. Latency to fall (in s) from the rotating bar was recorded. Mice were rested for 10 min after each trial, which was repeated three additional times per day for four consecutive days. A mouse's latency to fall for each day was recorded as the mean latency of the four consecutive trials (***p < 0.001 for all groups, one-way ANOVA).

### HITS-CLIP

HITS-CLIP cDNA libraries were generated as described previously with the following modifications (Charizanis et al, 2012). Quadriceps muscles were dissected from *Mbnl1*^+/+^, *Mbnl1*^−/−^ and *Mbnl2*^−/−^ mice (15–16 weeks of age), snap frozen and finely powdered in liquid N_2_ using a mortar and a pestle and crosslinked with UV-light using a Stratalinker 1800 (Stratagene). For cDNA library generation, WT and *Mbnl1*^−/−^ (*n* = 3 each) were analysed and the required RNA size distributions were generated using RNase A concentrations of 38.6 and 0.0386 U/ml for high and low RNase, respectively. Immunoprecipitation was performed using mAb 3B4 (3.25 μg/mg lysate) and cDNA libraries were generated and sequenced as described previously (Charizanis et al, 2012). Raw reads were filtered to remove low quality reads and filtered reads were aligned to the mouse reference genome (mm9) using the Burrows-Wheeler Aligner (BWA [m1]). Sequences <20 nt were discarded and unique CLIP tags were identified after computationally removing potential PCR duplicates as described (Charizanis et al, 2012) (Supporting Information Table S2).

### Accession number

HITS-CLIP data have been deposited in GEO under accession number GSE47794.

### The paper explained

**PROBLEM:**

Myotonic dystrophy (DM), the most common adult-onset muscular dystrophy, is characterized by multi-systemic symptoms and reduced life expectancy. A current disease model proposes that DM is caused by the expression of toxic microsatellite expansion RNAs that inhibit the RNA splicing function of the MBNL proteins resulting in the mis-expression of fetal isoforms in adult tissues. However, the loss of individual Mbnl proteins in mouse knockout models only partially recapitulates DM symptoms in skeletal muscle and the heart suggesting that the expression of other factors may be critical for DM pathogenesis.

**RESULTS:**

While loss of Mbnl1 expression leads to compensatory Mbnl2 upregulation in skeletal muscle and heart, combined loss of Mbnl1 and Mbnl2 leads to reduced lifespan, severe myotonia together with muscle weakness and wasting, cardiac conduction block and significant enhancement of DM-associated RNA splicing errors. These findings demonstrate that the major clinical manifestations of DM in specific tissues can be modelled by compound loss of Mbnl protein activity.

**IMPACT:**

RNA splicing errors in DM have been proposed as biomarkers for disease progression and therapeutic trials, and *Mbnl* compound knockout mice provide a model system to identify additional RNA mis-splicing events. This study also serves as model for ongoing investigations focused on other RNA-mediated diseases, including chromosome 9-linked frontotemporal dementia and amyotrophic lateral sclerosis (C9ORF72 FTD/ALS), and investigations on the roles of MBNL proteins in ES cell-specific alternative splicing regulation.

## Author contributions

Mouse lines were generated by KYL, KC, DF and HH. Experiments were designed and performed by KYL, ML, MM, RB and AM. Cardiac studies were planned and performed by ML, SAW, HA and HK and muscle analysis was carried out by KYL, ML, CMC and LPWR. The manuscript was prepared by KYL and MSS.

## For more information

1. OMIM for Myotonic Dystrophy:
2. http://www.omim.org/entry/160900
3. http://www.omim.org/entry/602668
4. Muscular Dystrophy Association:
5. http://www.mda.org
6. Myotonic Dystropy Foundation:
7. http://www.myotonic.org
8. Myotonic Dystrophy Support Group:
9. http://www.myotonicdystrophysupportgroup.org
10. Association Française contre les Myopathies:
11. http://www.afm-telethon.com
12. Treat-NMD Neuromuscular Network:
13. http://www.treat-nmd.eu

## Notes

As a service to our authors and readers, this journal provides supporting information supplied by the authors. Such materials are peer reviewed and may be re-organized for online delivery, but are not copy-edited or typeset. Technical support issues arising from supporting information (other than missing files) should be addressed to the authors.
