# Supplementary material for: Compound loss of muscleblind-like function in myotonic dystrophy
Source: EMBO Mol Med. 2013 Oct 8;5(12):1887–900. doi: 10.1002/emmm.201303275 (PMC3914532; doi:10.1002/emmm.201303275)
Supplement: Supplementary file 1 [file emmm0005-1887-sd1.pdf]

Compound loss of muscleblind-like function in myotonic dystrophy

Kuang-Yung Lee, Moyi Li, Mini Manchanda, Ranjan Batra, Konstantinos Charizanis, Apoorva Mohan, Sonisha A. Warren, Christopher M. Chamberlain, Dustin Finn, Hannah Hong, Hassan Ashraf, Hideko Kasahara, Laura P.W. Ranum and Maurice S. Swanson

Corresponding author: Maurice S. Swanson, University of Florida

Review timeline:

Submission date:	10 July 2013
Editorial Decision:	25 July 2013
Revision received:	13 August 2013
Editorial Decision:	27 August 2013
Revision received:	30 September 2013
Accepted:	06 September 2013

Transaction Report:

(Note: With the exception of the correction of typographical or spelling errors that could be a source of ambiguity, letters and reports are not edited. The original formatting of letters and referee reports may not be reflected in this compilation.)

Editor: Roberto Buccione

1st Editorial Decision 25 July 2013

Thank you for the submission of your manuscript to EMBO Molecular Medicine. We have now received comments from the three Reviewers whom we asked to evaluate your manuscript

You will see that all three Reviewers are generally supportive of you work, although they do raise issues that prevent us from considering publication at this time. I will not dwell into much detail, as the evaluations are detailed and self-explanatory. I would like, however, to mention that the main points are related to unclear statements or conclusions based on the data, the need for further quantification or extensions for some experiments, and requests for improved discussion of certain salient points. The comments appear nicely complementary and I am sure that they will ultimately help you produce an improved manuscript.

In conclusion, while publication of the paper cannot be considered at this stage, we would be pleased to consider a suitably revised submission, provided, however, that the Reviewers' concerns are fully addressed with additional experimental data where appropriate.

Please note that it is EMBO Molecular Medicine policy to allow a single round of revision only and that, therefore, acceptance or rejection of the manuscript will depend on the completeness of your responses included in the next, final version of the manuscript.

As you know, EMBO Molecular Medicine has a "scooping protection" policy, whereby similar findings that are published by others during review or revision are not a criterion for rejection. However, I do ask you to get in touch with us after three months if you have not completed your revision, to update us on the status. Please also contact us as soon as possible if similar work is published elsewhere.

I look forward to receiving your revised manuscript.

***** Reviewer's comments *****

Referee #1 (Comments on Novelty/Model System):

This paper addresses the important issue of gene function redundancy in myotonic dystrophy (DM). ds(CUG) hairpins have been found previously to sequester the human Muscleblind-like paralog proteins MBNL1 and MBNL2, effectively depleting these proteins from their normal cellular roles, but their potential overlapping functions were not adequately assessed. Whereas Mbnl1 KO mice reproduce some aspects of the DM pathology, others such as muscle weakness and wasting are not. The authors generate Mbnl1 KO mice in which the Mbnl2 dose has been halved and demonstrate that these mice develop cardinal features of DM disease including reduced lifespan, heart conduction block and muscle weakness. The analysis of the data is technically adequate, although not comprehensive, sincepotential brain function defects are not addressed. Potential compensation of Mbnl1 functions other than alternative splicing regulation are not analyzed either (e.g. miRNA biogenesis). On the other hand, the authors go to great lenghts to establish the functional compensation of Mbnl1 function by Mbnl2 in muscle by describing the enhanced binding of Mbnl2 to Mbnl1 normal targets in muscle transcripts. Novelty of the data is high. Individual KO mice were previously available, but the combined loss/reduction of Mbnl1/Mbnl2 function is described for the first time. The medical impact is regarded to as high. The study establishes that Mbnl2 can functionally compensate for Mbnl1, at least in skeletal and cardiac muscle, in vivo, and makes the important inference that CUG toxicity stems from sequestration of both MBNL1 and MBNL2 (which has been long suspected but not formally shown in vivo). The model system, mice genetically manipulated to lack functional Mbnl1/Mbnl2 genes, is definitely the best possible.

Referee #1 (Remarks):

This paper addresses the important issue of gene function redundancy in myotonic dystrophy (DM). ds(CUG) hairpins have been found previously to sequester the human Muscleblind-like paralog proteins MBNL1 and MBNL2, effectively depleting these proteins from their normal cellular roles, but their potential overlapping functions were not adequately assessed in vivo. Whereas Mbnl1 KO mice reproduce some aspects of the DM pathology, others such as muscle weakness and wasting are not. The authors generate Mbnl1 KO mice in which the Mbnl2 dose has been halved and demonstrate that these mice develop cardinal features of DM disease including reduced lifespan, heart conduction block and muscle weakness. The papers has many strenghts and this reviewer finds it suitable for publication with the following recommendations:
1.- The authors adequately address the issue of functional compensation of Mbnl1 by Mbnl2 in skeletal and cardiac tissue, however, not much is said as for a similar compensation in the brain, where Mbnl1 is also expressed. Similarly, only the role of Mbnl1 in regulating alternative splicing of muscle transcripts is analyzed whereas it has been shown that MBNL1 also regulates miRNA biogenesis (miR1) or RNA stability. Whereas it is reasonable that the study was not comprehensive, it would be desirable that the authors more carefully discuss these issues in the discussion section.
2.- Throughout the paper several examples of missplicing events are shown. To discard a global effect on alternative splicing regulation, a few examples of unchanged use of alternative exons would be appreciated.
3.- Images in Fig. 2A are assumed to be representative, however, it would be relatively easy to quantify the increase in histophatology signs (central nuclei, for example). Same applies to Supp. Fig. S3A, where fibrosis can be probably quantified.
4.- Fig. 3B, quantification of Tnnt3 fetal exon inclusion, is a bit puzzling and may require some additional explanation since the banding pattern is quite different in Mbnl1-/- and Mbnl1 -/- Mbnl2 +/-. In the same figure, significance (asterisks) in Cacna1s (WT vs Mbnl1 -/-) is surprisingly high given the apparent small difference between both genotypes. Same with Tnnt3EF.
5.- It is unclear whether the results statement that "...2-9-fold increase in Mbnl2 binding to previously documented Mbnl1 skeletal muscle RNA targets" is solely based in the data shown in Fig. 6B. In particular, only 10 transcripts are shown with increased binding of Mbnl2. Are there more? All differences in Mbnl2 binding to RNA shown in panel (B) are relevant? By which criteria?

6.- Model figure may require some additional explanations because the proposal that relatively short CUG expansions preferentially sequester MBNL1, and only secondarily, when expansions are long, sequesters MBNL2 seems new (please cite supporting data).

Minor comments:
1.- Please revise proper use of unit abbreviations (s, nor sec)
2.- in page 18 a "stained" seems to be required in the sentence "...of tibialis anterior muscle were with..."
3.- Fig 1D legend. n=10 for both genetypes, but three genotypes are shown

Referee #2 (Comments on Novelty/Model System):

Studies of single Mbnl1-/- KO and single Mbnl2-/- KO mouse models have already learned us a lot about involvement of Mbnl biology in DM pathology. Here the generation of compound mutants is a next logical step.

Referee #2 (Remarks):

In this manuscript Lee and co-authors (with dr. M. Swanson as senior author) present new evidence in support of a role for members of the MBNL family in DM pathobiology. By crossing-in of two existing mouse models the authors have generated Mbnl1-/-;Mbnl2+/- compound knockout mice. Solid molecular, histological and physiological-functional evidence is presented to demonstrate that profound alterations occur in the alternative splice patterns for various Mbnl hnRNA targets in both muscle and heart tissue, with concomitant effects on NMJ structures, muscle strength and mobility and cardiac function and tissue morphology.

Strength: This is a well-written paper. Although the study does not provide truly new mechanistic insight in Mbnl's central role in DM pathophysiology and although the manuscript's contents is mainly of descriptive nature, the authors managed to provide important new information on the possible role of Mbnl factors in DM1 disease manifestation in muscle and heart. Experimental approaches are sound and overall quality of the figures is excellent.

Weakness: On the last pages of the submission (pg. 25-26) the authors explain once more the problem, the results and impact of this study. Here, they briefly mention that the compound mouse model may be a valuable asset for further study of the newly disclosed role of MBNL proteins as well as of ES-cell specific alternative splice regulators (see Ref. Han et al. (2013) Nature 498, 241). Exactly these points, the difficulty to distinguish between primary and secondary developmental effects, which is clearly due to the complexity of biological functions of MBNL proteins should have been discussed more thoroughly in the Introduction and in the Discussion sections.

I therefore recommend that the following aspects be addressed in a revised version of this manuscript:
a. In the introduction, the authors should mention that multiple splice isoforms do exist for each of the three MBNL proteins, Mbnl1-3. Moreover, work by the groups of Drs. Ishiura and Reddy has demonstrated that binding to (C(C)UG hairpins maybe isoform specific.
b. Fig. 1 shows an increase in the level of Mbnl2 in Mbnl1 KO muscles. Normally, in development Mbnl2 expression is progressively lost upon terminal muscle differentiation in vivo. Throughout the paper the authors consistently speak of "upregulation" of Mbnl2 in MBnl1 KO muscles, but they should consider (and discuss!) the possibility that the lack of Mbnl1 creates an embryonal/ES cellular phenotype, whereby Mbnl2 gene expression of both alleles in Mbnl1-/- mice and the remaining allele in Mbnl1-/-;Mbnl2+/- mice is maintained at a higher level purely because this is appropriate for the immature character of the muscle. So could it be the lack of gene repression for Mbnl2, not an adaptation with up-regulation caused by loss of "redundant" gene family members that is involved? Theoretical question: Would allelic loss of Mbnl2 activity also have synergistic effects if induced later in development (this question can only be studied in Cre-tailored KO models). From the strength of signals for Mbnl2 on Western blots shown in Fig. 1A, one gets the impression that 3-5 fold more Mbnl2 is present in compound mutant muscle than in wt muscle. At the same time it appears that there is equal or at most only 2-fold more Mbln2 in the heart of double

mutant mice and that Mbnl2 remains at similar levels in brains of Mbnl1-/-;Mbnl2+/- and wt mice. Is a different outcome for (residual) Mbnl2 expression level seen if these tissues are analyzed at an earlier or a later time point?
c. How do the authors explain the early death and diminished body weight in compound mutant mice? Is there abnormal presence of satellite/muscle stem cells in the Mbnl1-/-;Mbnl2+/- animals?
d. In the supplementary figure the authors demonstrate altered splicing/mis-splicing of exons 6 and 8 in MBNL2 pre-mRNA. The consequences of this missplicing are not well discussed. What effects does presence/absence of exon6/8 encoded protein domains in Mbnl2 have on normal recognition of exon-intron junctions and on recognition of the C(C)UG hairpin structure? In other words, are some of the effects seen caused by general upregulation of Mbnl2 isoforms, or specifically caused by upregulation of these distinct splice isoforms (see remarks made under (b); in heart there is almost wt level of protein, still effects are seen. Could this be due to another splice isoform composition for Mbnl2?

Referee #3 (Comments on Novelty/Model System):

Myotonic dystrophy is a genetic disease caused by expansion of C(C)TG repeats in DNPK (DM1) or CNBP (DM2) genes. Studies from several labs have demonstrated that expanded C(C)TG repeats affect the expression/activity of several RNA-binding proteins (Mbnl1, Mbnl2, Mbnl3, CUGBP1 and ETR3-like (CELF), hnRNP H and STAU1). While transgenic mice overexpressing polyCUG display a variety of DM symptoms, KO mice for a single polyCUG-binding protein display only a subset of these. In particular, Mbnl1 KO mice display myotonia, cataracts and a mild myopathy, while Mbnl2 KO mice display mainly brain phenotypes. Since Mbnl2 is expressed mainly in brain, the above results were interpreted as if polyCUG in DM might affect different proteins in different tissues. In this manuscript Lee et al. present an alternative explanation. The study started from the observation that Mbnl2 levels are significantly increased in Mbnl1 KO mice suggesting that Mbnl2 could compensate for Mbnl1 loss. To assess this, they investigated the phenotype and molecular features of compound Mbnl1 -/-;Mbnl2 +/- (compound KO) mice. Interestingly, compound KO mice develop more severe and more precocious skeletal muscle defects. Moreover, they develop cardiac defects reminiscent of DM that are not present in Mbnl1 or Mbnl2 single KO mice. Significantly, these phenotypes are accompanied by more severe alternative-splicing changes in transcripts aberrantly spliced in DM. To complete nicely the story, the authors performed HITS-CLIP for Mbnl2 in WT and Mbnl1 KO mice finding that Mbnl2 is signicantly more recruited to affected transcripts in Mbnl1 KO mice.
This is a very well done study that provides a better understanding of DM pathology, additional splicing targets and an intersting new animal model of the disease.

Referee #3 (Remarks):

I have only minor comments:

- Figure 1A. It would be useful to provide (as a Supplementary Figure) evidence that the other polyCUG-binding proteins (Mbnl3, CUGBP1 and ETR3-like (CELF), hnRNP H and STAU1) are not affected in Mbnl1 KO mice to support the specificity of the Mbnl2 increased expression in Mbnl1 KO mice. This also considering that some of them (for example Mbnl3) have been reported to be increased in DM patients.
- Figure 3A. There is either a problem of not equal loading or of differential Clcn1 transcript stability in the different samples. Please explain.
- Figure 3B. It would be useful to provide (as a Supplementary Figure) images supporting the splicing changes of Cacna1s, Serca1, Ryr1 E83, and Nfix.
- Figure 5B. It would be useful to provide (as a Supplementary Figure) images supporting the splicing changes of Cacna1s, Spag9, Mbnl1 and Arhgef7.
- Figure 6D. To support this hypothesis, authors could investigate relative Mbnl1/2 levels in poly(CUG) mice at various time points.

1st Revision - authors' response 13 August 2013

We would like to thank the Referees for their complimentary remarks as well as the insightful and helpful comments on our manuscript entitled '*Compound loss of muscleblind-like function in myotonic dystrophy*'. As detailed below, we have responded to the all of their concerns and have included an additional figure (new Figure 6) requested by Referee #2 which describes a muscle-specific knockout experiment. We feel these modifications have resulted in a significantly improved manuscript.

Referee #1

1. The authors adequately address the issue of functional compensation of Mbnl1 by Mbnl2 in skeletal and cardiac tissue, however, not much is said as for a similar compensation in the brain, where Mbnl1 is also expressed. Similarly, only the role of Mbnl1 in regulating alternative splicing of muscle transcripts is analyzed whereas it has been shown that MBNL1 also regulates miRNA biogenesis (miR1) or RNA stability. Whereas it is reasonable that the study was not comprehensive, it would be desirable that the authors more carefully discuss these issues in the discussion section.

We agree that the issues of MBNL activities in the brain and in muscle miRNA biogenesis and RNA stability were not adequately discussed. We have revised the Discussion section (Pg. 17) to address these important issues.

2. Throughout the paper several examples of missplicing events are shown. To discard a global effect on alternative splicing regulation, a few examples of unchanged use of alternative exons would be appreciated.

Examples are now included in **Figure S1F**. In addition, we have modified the text (Pg. 9, 2nd to last sentence) to highlight previously published results that demonstrate that many characterized alternative splicing events are not regulated by the Mbnl proteins (e.g., see Kanadia et al., *Science* 302:1978-1980 Fig. 3C).

3. Images in Fig. 2A are assumed to be representative, however, it would be relatively easy to quantify the increase in histopathology signs (central nuclei, for example). Same applies to Supp. Fig. S3A, where fibrosis can be probably quantified.

These examples are now included in the **Figure 6C** and **Figure S3B**.

4. Fig. 3B, quantification of Tnnt3 fetal exon inclusion, is a bit puzzling and may require some additional explanation since the banding pattern is quite different in Mbnl1-/- and Mbnl1 -/- Mbnl2 +/-. In the same figure, significance (asterisks) in Cacna1s (WT vs Mbnl1 -/-) is surprisingly high given the apparent small difference between both genotypes. Same with Tnnt3EF.

We thank the Referee for catching these errors and the placement of these brackets/asterisks has been corrected.

5. It is unclear whether the results statement that "...2-9-fold increase in Mbnl2 binding to previously documented Mbnl1 skeletal muscle RNA targets" is solely based in the data shown in Fig. 6B. In particular, only 10 transcripts are shown with increased binding of Mbnl2. Are there more? All differences in Mbnl2 binding to RNA shown in panel (B) are relevant? By which criteria?

The Referee is correct and this statement was incomplete. We have added several sentences (Pg. 14) that state the number of genes showing increased Mbnl2 binding and the functional categories that are represented.

6. Model figure may require some additional explanations because the proposal that relatively short CUG expansions preferentially sequester MBNL1, and only secondarily, when expansions

are long, sequesters MBNL2 seems new (please cite supporting data).

We have modified the Discussion text (Pg. 18) to provide a clearer explanation for this model.

Minor comments:

1. Please revise proper use of unit abbreviations (s, nor sec).

Corrected throughout the manuscript.

2. Page 18 a "stained" seems to be required in the sentence "...of tibialis anterior muscle were with..."

Corrected as requested.

3. Fig 1D legend. n=10 for both genotypes, but three genotypes are shown
Corrected.

Referee #2

a. In the introduction, the authors should mention that multiple splice isoforms do exist for each of the three MBNL proteins, Mbnl1-3. Moreover, work by the groups of Drs. Ishiura and Reddy has demonstrated that binding to (C(C)UG hairpins maybe isoform specific.

As requested, a new sentence has been added to the Introduction (Pg. 3 and 4) that discusses the various splice isoforms of the three MBNL proteins and the appropriate references have been added.

b. Fig. 1 shows an increase in the level of Mbnl2 in Mbnl1 KO muscles. Normally, in development Mbnl2 expression is progressively lost upon terminal muscle differentiation in vivo. Throughout the paper the authors consistently speak of "upregulation" of Mbnl2 in Mbnl1 KO muscles, but they should consider (and discuss!) the possibility that the lack of Mbnl1 creates an embryonal/ES cellular phenotype, whereby Mbnl2 gene expression of both alleles in Mbnl1-/- mice and the remaining allele in Mbnl1-/-;Mbnl2+/- mice is maintained at a higher level purely because this is appropriate for the immature character of the muscle. So could it be the lack of gene repression for Mbnl2, not an adaptation with up-regulation caused by loss of "redundant" gene family members that is involved? Theoretical question: Would allelic loss of Mbnl2 activity also have synergistic effects if induced later in development (this question can only be studied in Cre-tailored KO models). From the strength of signals for Mbnl2 on Western blots shown in Fig. 1A, one gets the impression that 3-5 fold more Mbnl2 is present in compound mutant muscle than in wt muscle. At the same time it appears that there is equal or at most only 2-fold more Mbln2 in the heart of double mutant mice and that Mbnl2 remains at similar levels in brains of Mbnl1-/- ;Mbnl2+/- and wt mice. Is a different outcome for (residual) Mbnl2 expression level seen if these tissues are analyzed at an earlier or a later time point?

The Referee makes a number of valid points and we agree that an alternative explanation for our observations is that elevated Mbnl2 levels in Mbnl1 KO mice simply reflects muscle immaturity. We now discuss this possibility (Results, Pg. 12) and also have added the reference to the recently published Han et al paper. In addition, we have addressed the 'theoretical question' posed by the Referee and included results from a new conditional model (*Mbnl*1-/-; *Mbnl2*cond/cond; *Myog-cre*+) in which Mbnl2 expression is eliminated only in the skeletal muscle compartment at a later developmental period compared to the constitutive KO (see Results Pg. 12, new section entitled 'Mbnl conditional compound loss of function model for DM') and **new Figure 6**. For the question of Mbnl2 expression at different time points, we have examined the 16-20 weeks of age range and not later time points because the maximum lifespan of this line is 23 weeks of age; early time points have not been assessed.

c. How do the authors explain the early death and diminished body weight in compound mutant mice? Is there abnormal presence of satellite/muscle stem cells in the Mbnl1-/-;Mbnl2+/-

animals?.

We now discuss that lower body weight has also been noted in *Mbnl2* KO (Charizanis et al, 2012) and DMSXL polyCUG homozygous transgenic mice (Gomes-Pereira et al, 2007) and since the *Mbnl*1-/-; *Mbnl2*cond/cond; *Myog-cre*+ mice are also small while *Mbnl1* KOs are not, we mention that expression of Mbnl proteins in the embryonic muscle compartment may have important implications for normal body size development (Discussion, Pg 17). We do not currently know the reason for the early death and we have not assayed whether the satellite cell population is abnormal in *Mbnl1-/-; Mbnl2+/-* mice.

d. In the supplementary figure the authors demonstrate altered splicing/mis-splicing of exons 6 and 8 in MBNL2 pre-mRNA. The consequences of this missplicing are not well discussed. What effects does presence/absence of exon6/8 encoded protein domains in Mbnl2 have on normal recognition of exon-intron junctions and on recognition of the C(C)UG hairpin structure? In other words, are some of the effects seen caused by general upregulation of Mbnl2 isoforms, or specifically caused by upregulation of these distinct splice isoforms (see remarks made under (b); in heart there is almost wt level of protein, still effects are seen. Could this be due to another splice isoform composition for Mbnl2?

We agree that the results on altered splicing of Mbnl2 exons 6 and 8 was not adequately discussed and this have now been corrected in the Results section (Pg. 7, end of 1st paragraph).

Referee #3 (only minor comments)

1. Figure 1A. It would be useful to provide (as a Supplementary Figure) evidence that the other polyCUG-binding proteins (Mbnl3, CUGBP1 and ETR3-like (CELF), hnRNP H and STAU1) are not affected in Mbnl1 KO mice to support the specificity of the Mbnl2 increased expression in Mbnl1 KO mice. This also considering that some of them (for example Mbnl3) have been reported to be increased in DM patients.

This information has been added in **Figure S1A** (Celf1/Cugbp1 was not included but Fig S1 legend notes that previous work has shown that this protein is not upregulated in *Mbnl1* KO mice).

2. Figure 3A. There is either a problem of not equal loading or of differential Clcn1 transcript stability in the different samples. Please explain.

The Clcn1 pattern is highly reproducible (e.g., see Charizanis et al., *Neuron* 75:437-450 Figure 1D) and is likely the result of differences RNA stability. This is now noted in the Figure 3A legend.

3. Figure 3B. It would be useful to provide (as a Supplementary Figure) images supporting the splicing changes of Cacna1s, Serca1, Ryr1 E83, and Nfix.

These additional images have been included in **Figure S1E**.

4. Figure 5B. It would be useful to provide (as a Supplementary Figure) images supporting the splicing changes of Cacna1s, Spag9, Mbnl1 and Arhgef7.

These additional images have been included in **Figure S3E**.

5. Figure 6D. To support this hypothesis, authors could investigate relative Mbnl1/2 levels in poly(CUG) mice at various time points.

This is an excellent suggestion but we feel this analysis in transgenic polyCUG mice is beyond the scope of this paper.

2nd Editorial Decision 27 August 2013

Thank you for the submission of your revised manuscript to EMBO Molecular Medicine. We have now received the enclosed reports from the Reviewers that were asked to re-assess it. As you will see the reviewers are now supportive. Before I can accept your manuscript for publication, however, there remain a few pending issues that require your action:

1) While performing our pre-publishing quality control and image screening routines, we noticed an issue with Figure 1A. Specifically, assembly of the central blot appears the result of splicing in of two different components (and with clearly heterogeneous resolution). I am sure you will understand that this issue prevents us from moving forward with your manuscript until satisfactory clarification is provided. To this end, we ask you to please send us a detailed explanation together with the source data for this experiment.

2) The current resolution of Figure 4A is poor and should be improved

3) As per our Author Guidelines, the description of all reported data that includes statistical testing must state the name of the statistical test used to generate error bars and P values, the number (n) of independent experiments underlying each data point (not replicate measures of one sample), and the actual P value for each test (not merely 'significant' or 'P < 0.05').

Please submit your revised manuscript toghether with accessory information, within two weeks. I look forward to seeing a revised form of your manuscript as soon as possible.

***** Reviewer's comments *****

Referee #1 (Comments on Novelty/Model System):

This paper addresses the important issue of gene function redundancy in myotonic dystrophy (DM). ds(CUG) hairpins have been found previously to sequester the human Muscleblind-like paralog proteins MBNL1 and MBNL2, effectively depleting these proteins from their normal cellular roles, but their potential overlapping functions were not adequately assessed. Whereas Mbnl1 KO mice reproduce some aspects of the DM pathology, others such as muscle weakness and wasting are not. The authors generate Mbnl1 KO mice in which the Mbnl2 dose has been halved and demonstrate that these mice develop cardinal features of DM disease including reduced lifespan, heart conduction block and muscle weakness. The analysis of the data is technically adequate, although not comprehensive, since potential brain function defects are not addressed. Potential compensation of Mbnl1 functions other than alternative splicing regulation are not analyzed either (e.g. miRNA biogenesis). On the other hand, the authors go to great lenghts to establish the functional compensation of Mbnl1 function by Mbnl2 in muscle by describing the enhanced binding of Mbnl2 to Mbnl1 normal targets in muscle transcripts. Novelty of the data is high. Individual KO mice were previously available, but the combined loss/reduction of Mbnl1/Mbnl2 function is described for the first time. The medical impact is regarded to as high. The study establishes that Mbnl2 can functionally compensate for Mbnl1, at least in skeletal and cardiac muscle, in vivo, and makes the important inference that CUG toxicity stems from sequestration of both MBNL1 and MBNL2 (which has been long suspected but not formally shown in vivo). The model system, mice genetically manipulated to lack functional Mbnl1/Mbnl2 genes, is definitely the best possible.

Referee #1 (Remarks):

The authors have adequately addressed all concerns raised by this reviewer

Referee #2 (Comments on Novelty/Model System):

In the revised version of the manuscript, most issues raised by this reviewer were now adequately addressed. Unfortunately, from the results provided it is still difficult to distinguish between primary and secondary effects and obtain a reliable estimate of the effects of muscle immaturity/regeneration. Follow up studies therefore will remain necessary.

Referee #2 (Remarks):

In the revised version of the manuscript, most issues raised by this reviewer were now adequately addressed. Effects on MBNL2 isoform expression during the course of development and effects of muscle regeneration certainly need further attention in follow-up studies.

Referee #3 (Comments on Novelty/Model System):

The authors have built a very solid story convincingly showing Mbnl1/Mbnl2 compensation in vivo. Results from this study are relevant for the production of reliable animal models of DM, clarifying the molecular pathogenesis and identifying possible disease biomarkers/therapeutic targets.

Referee #3 (Remarks):

The authors have fully addressed my concern.

2nd Revision - authors' response 30 September 2013

Thank you for the supportive letters from the Reviewers and your editorial assistance. I have addressed the three pending issues as noted below:

1) While performing our pre-publishing quality control and image screening routines, we noticed an issue with Figure 1A. Specifically, assembly of the central blot appears the result of splicing in of two different components (and with clearly heterogeneous resolution). I am sure you will understand that this issue prevents us from moving forward with your manuscript until satisfactory clarification is provided. To this end, we ask you to please send us a detailed explanation together with the source data for this experiment.

Thank you for noticing this error. We have replaced the central blot with the correct version and I have included a pdf of the source data.

2) The current resolution of Figure 4A is poor and should be improved

This is the highest resolution data that we obtained from this 4.7T MRI dataset and is comparable to similar published results (e.g., Schips et al, Cardiovas Res 91:587-597).

3) As per our Author Guidelines, the description of all reported data that includes statistical testing must state the name of the statistical test used to generate error bars and P values, the number (n) of independent experiments underlying each data point (not replicate measures of one sample), and the actual P value for each test (not merely 'significant' or 'P < 0.05').

All threshold p values (e.g., $p < 0.05$) have now been replaced with actual p values and the number (n) of independent experiments and the statistical tests used are now noted in the relevant figure legends.

© EMBO
