# Supplementary material for: Compound loss of muscleblind-like function in myotonic dystrophy
Source: EMBO Mol Med. 2013 Oct 8;5(12):1887–900. doi: 10.1002/emmm.201303275 (PMC3914532; doi:10.1002/emmm.201303275)
Supplement: Supplementary file 2 [file emmm0005-1887-sd2.pdf]

Supporting Information

Lee *et al.*, Compound loss of muscleblind-like function in myotonic dystrophy

Table of Contents:

Supporting Information Figure S1: Analysis of *Mbnl1*; *Mbnl2* DKO mice

Supporting Information Figure S2: Mbnl2 mis-splicing in *Mbnl1*$^{-/-}$; *Mbnl2*$^{+/-}$ muscle and heart

Supporting Information Figure S3: Cardiac dysfunction in *Mbnl1*$^{-/-}$; *Mbnl2*$^{+/-}$ KO mice

Supporting Information Table S1: Primers for Mbnl1 and Mbnl2 compound knockout analysis

Supporting Information Table S2: HITS-CLIP analysis (Excel file)

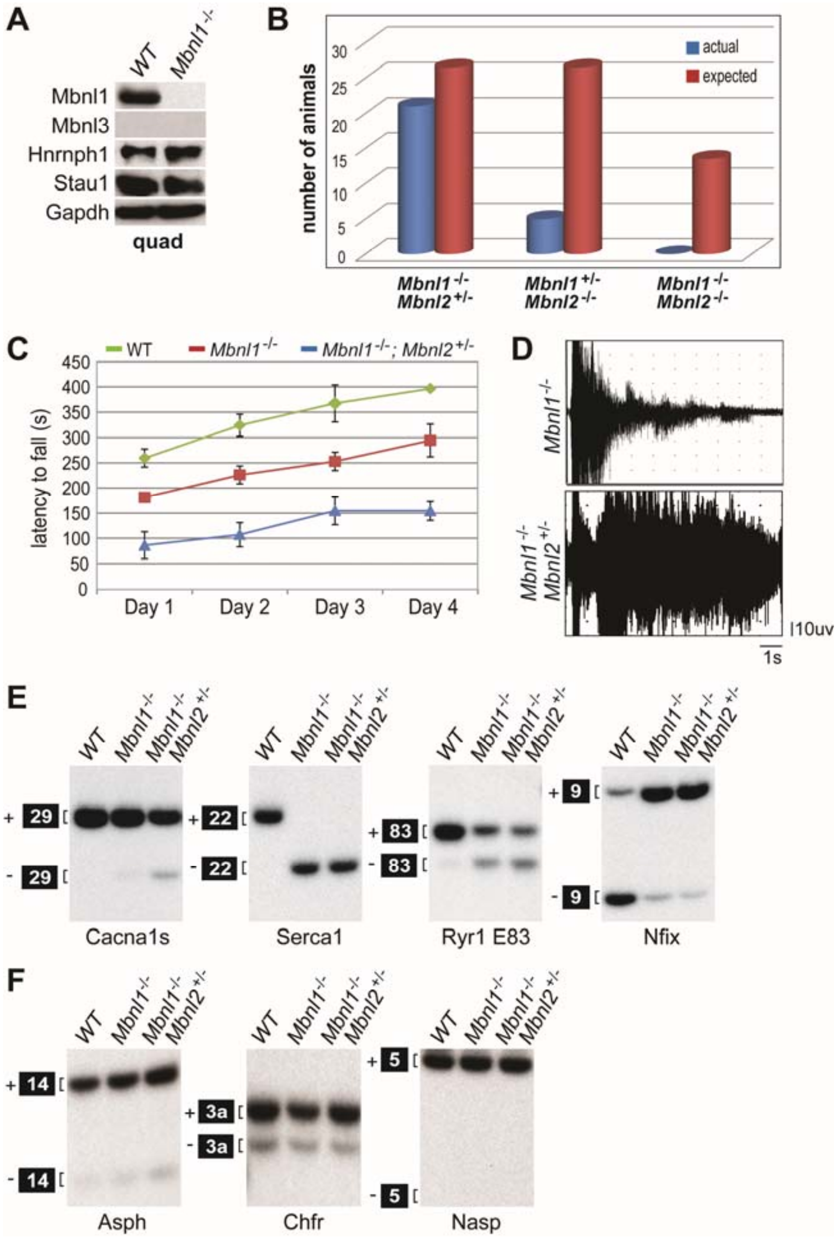

Supporting Information Figure S1

Analysis of *Mbnl1*; *Mbnl2* DKO mice. (**A**) Immunoblots of WT and *Mbnl1* KO (*Mbnl1*$^{-/-}$) adult quadriceps (quad) showing that major changes in Mbnl3, Hnrnph1 and Stau1 protein levels do not occur following Mbnl1 loss. Celf1 levels are also not affected in *Mbnl1* KOs (Kanadia et al, 2003). (**B**) *Mbnl1*$^{+/\Delta E3}$ X *Mbnl2*$^{+/\Delta E2}$ crosses resulted in nearly the expected number (red) of *Mbnl1*$^{-/-}$; *Mbnl2*$^{+/-}$ mice (blue, actual number obtained), lower than expected numbers of *Mbnl1*$^{+/-}$; *Mbnl2*$^{-/-}$ KOs and no viable *Mbnl1*$^{-/-}$; *Mbnl2*$^{-/-}$ DKO mice. (**C**) Rotarod analysis of *Mbnl1*$^{+/+}$ (WT, green), *Mbnl1*$^{-/-}$ KO (red) and *Mbnl1*$^{-/-}$; *Mbnl2*$^{+/-}$ mice during the 4-day testing regimen. (**D**) Representative electromyography for *Mbnl1* KO (*Mbnl1*$^{-/-}$) versus *Mbnl1*$^{-/-}$; *Mbnl2*$^{+/-}$ mice. (**E**) RT-PCR splicing analysis of WT, *Mbnl1*$^{-/-}$ and *Mbnl1*$^{-/-}$; *Mbnl2*$^{+/-}$ KO mice for Cacna1s, Serca1, Ryr1 exon 83 and Nfix. (**F**) RT-PCR splicing analysis demonstrating that loss of Mbnl proteins does not result in a global effect on splicing regulation with no significant changes for Asph, Chfr or Nasp.

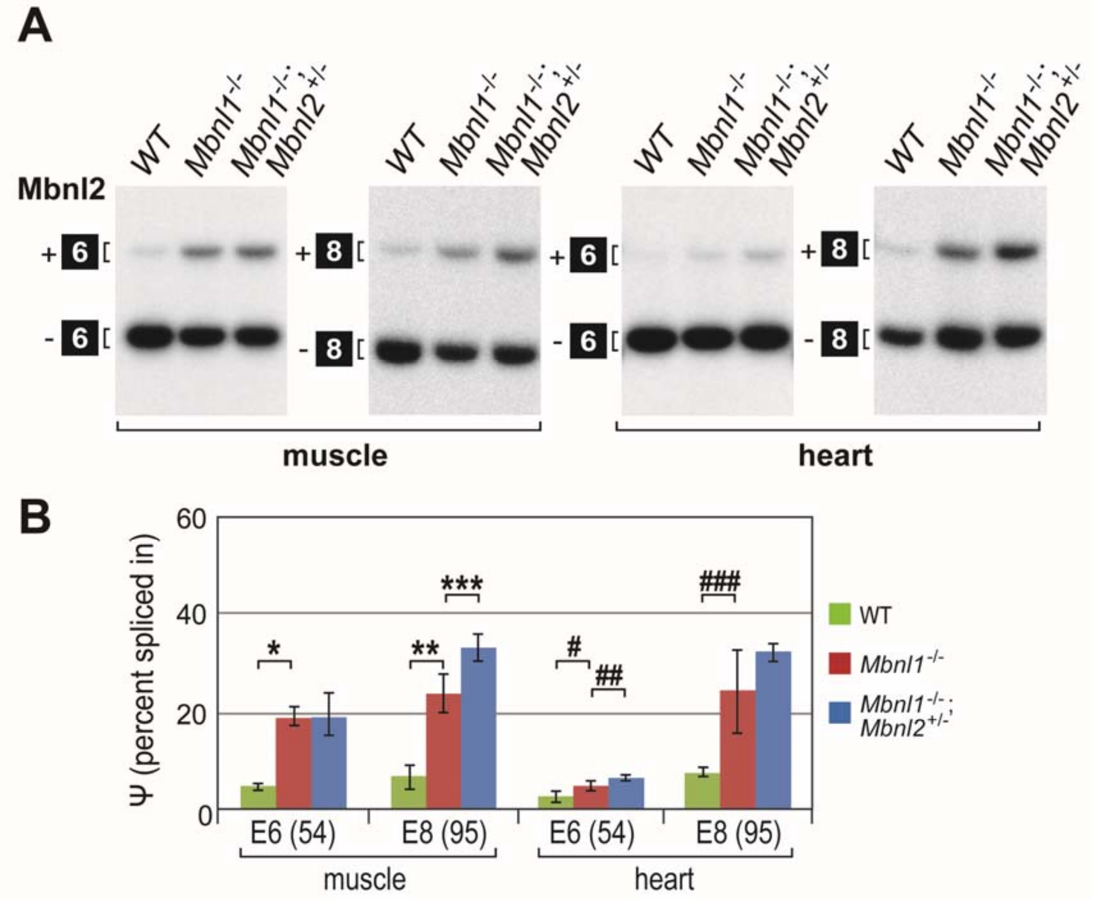

Supporting Information Figure S2

Mbnl2 mis-splicing in *Mbnl1*$^{-/-}$; *Mbnl2*$^{+/-}$ muscle and heart. (**A**) RT-PCR splicing assay showing an increase in mis-splicing of Mbnl2 exons 6 (54 nt) and 8 (95 nt) in *Mbnl1*$^{-/-}$; *Mbnl2*$^{+/-}$ versus WT and *Mbnl1*$^{-/-}$ KOs. (**B**) Percent spliced in values (ψ) for Mbnl2 exons 6 and 8 in muscle and heart for WT (green), *Mbnl1*$^{-/-}$ (red) and *Mbnl1*$^{-/-}$; *Mbnl2*$^{+/-}$ (blue) (n=4, *p = 0.00025, **p = 0.00168, ***p = 0.016, #p = 0.037, ##p = 0.019, ###p = 0.0235, Student's t-test).

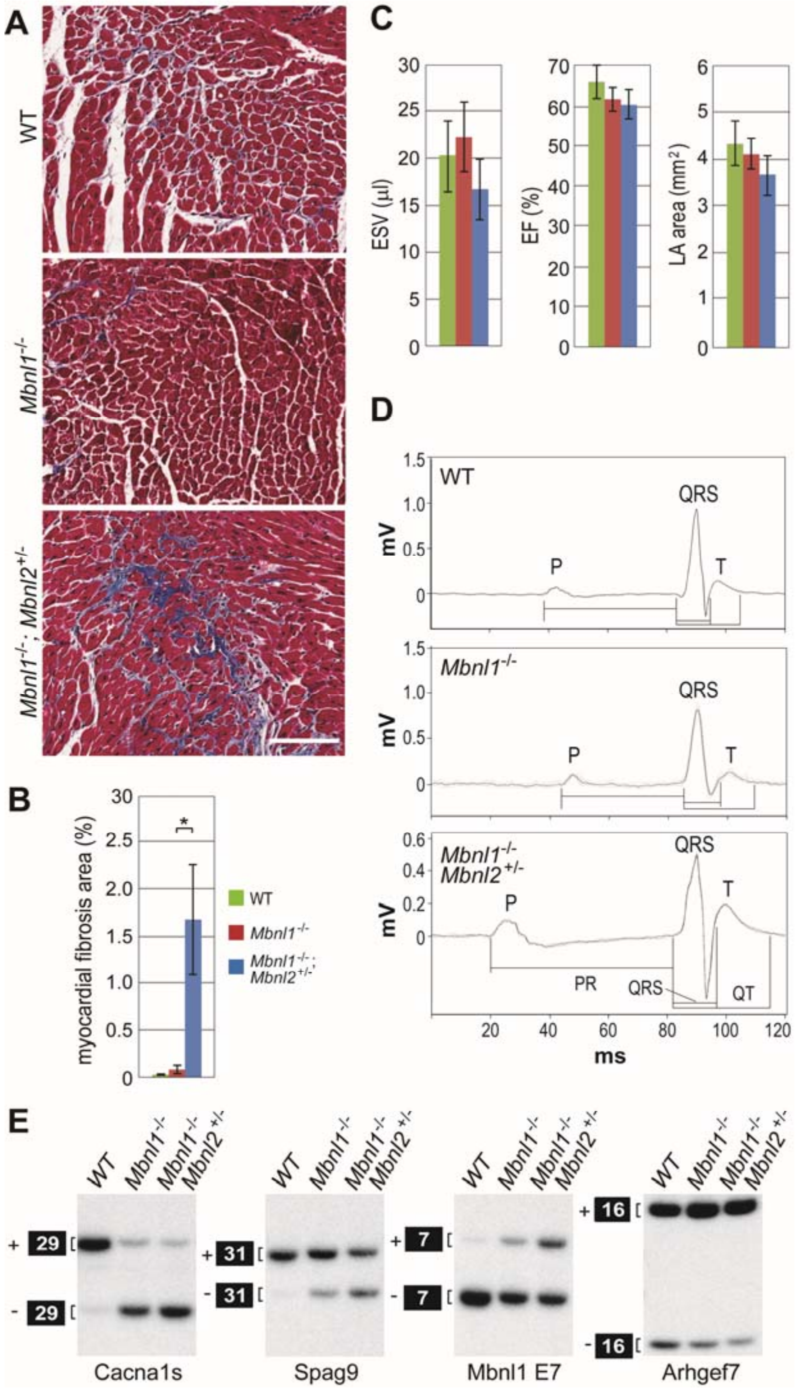

Supporting Information Figure S3

Cardiac dysfunction in *Mbnl1*$^{-/-}$; *Mbnl2*$^{+/-}$ KO mice. (**A**) Increased focal fibrosis (Masson's trichrome staining, blue) in left ventricular wall in *Mbnl1*$^{-/-}$; *Mbnl2*$^{+/-}$ KO versus WT and *Mbnl1*$^{-/-}$ hearts (scale bar represents 100 µm). (**B**) Comparison of myocardial fibrosis total area in WT (green), *Mbnl1*$^{-/-}$ (red) and *Mbnl1*$^{-/-}$; *Mbnl2*$^{+/-}$ (blue) KOs. (n = 3, *p = 0.0481, Student's t-test). (**C**) End systolic volume (ESV), ejection fraction (EF) and left atrial area (LA area) were not significantly different between WT (green), *Mbnl1*$^{-/-}$ (red) and *Mbnl1*$^{-/-}$; *Mbnl2*$^{+/-}$ (blue) KO hearts (n=5). (**D**) ECG analysis showing prolongation of PR, QRS and QT intervals in *Mbnl1*$^{-/-}$; *Mbnl2*$^{+/-}$ KO versus WT mice. (**E**) RT-PCR splicing analysis of WT, *Mbnl1*$^{-/-}$ and *Mbnl1*$^{-/-}$; *Mbnl2*$^{+/-}$ KO mice for Cacna1s, Spag9, Mbnl1 exon 7 (54 nt) and Arhgef7.

Supporting Information Table S1:

Primers for *Mbnl1* and *Mbnl2* compound knockout analysis

Gene	Exon	Exon (nt)	Forward primer (name)	Reverse primer (name)
Clcn1	7a	79	ttcacatcgccagcatctgtgc (MSS2788)	cacggaacacaaaggcactgaatgt (MSS1653)
Ryr1	70	15	gcgtgaagaacagaacttcgtggtc (MSS3890)	cttggtgcgttcctgatctgagc (MSS3891)
Cacna1s	29	57	gagatccttggaatgtgtttgacttcct (MSS4433)	ggttcagcagcttgaccagtctcat (MSS4434)
Serca1	22	41	gctcatggtcctcaagatctcac (MSS2761)	gggtcagtgcctcagctttg (MSS2762)
Bin1	11	45	cagaacctcaatgatgtcctggtca (MSS4578)	ctctggctcatggttcactctgatct (MSS4579)
Ryr1	83	18	cttcgagaggcagaacaaggcag (MSS3892)	aacaggtcctgtgtgaactcgtcatc (MSS3893)
Tnnt3	9	39	tctgacgaggaaactgaacaag (MSS1677)	tgtcaatgagggcttggag (MSS1678)
Nfix	8	123	ggagagtccagtagatgatgtgttctatcct (MSS4306)	ggatgatggacgtggaagggaa (MSS4307)
Tnnt2	4,5,6	11,12,30	gccgaggaggtggtggaggagta (MSS4288)	gtctcagcctcaccctcaggctca (MSS4289)
Ryr2	4,5	21,15	cggacctgtctatctgcacctttgt (MSS4284)	cataccactgtaggaatggcgtagca (MSS4285)
Sorbs1	25	168	ccagctgattacttggagtccacagaag (MSS2939)	gttcaccttcataccagttctggtcaatc (MSS2941)
Tmem63b	4	39	ctggctctggacttcatgtgctttc (MSS2981)	gagacggaggtgagacgctcatacc (MSS2982)
Spag9	31	39	ggactggaaatggtgtcattatctccat (MSS4303)	gggactgccacaaagaatttcacag (MSS4304)
Mbnl1	7	54	ggctgcccaataccaggtcaac (MSS4350)	gggagaaatgctgtatgctgctgtaa (MSS4351)
Afhgef7	16	225	gcagacgaaggtcacatctgtgagc (MSS2983)	cacagcgaactcctcgtccgaag (MSS2984)
Mbnl2	6	54	tcaccctcctgcacacttgcag (MSS3950)	ctctttggtaagggatgaagagcacc (MSS4021)
Mbnl1	9	95	tattgtgcatgacacccgctacaagt (MSS4622)	tgtgacgacagctctacatctgggtaa (MSS4623)
Mbnl2	8	95	cgtcttgcactaccagcaggctc (MSS3951)	gacatagcagaactagccttagggttgtg (MSS3953)
Csda	6	207	atggagttcctgtagaagggagtcgctat (MSS4733)	aacctcgggcggtaagtcggat (MSS4734)
Abi2	7	183	gcagtggaggaagtcacccaagtagt (MSS4636)	gaaggcttgtttgtgacgttatggaa (MSS4667)
Lnp	13	83	gctcaactggtcccacgctactagaa (MSS4713)	cttggcttgtctgctctgtaactggtatt (MSS4714)
Asph	14	48	catcgcagaaccatccaaatgaca (MSS4604)	tggttcatcatagtcttggtgtcttacttcc (MSS4605)
Chfr	3a	73	gtcctatggagctacacggggaaga (MSS4609)	gtgtcttccagtgtcacctcaccagat (MSS4601)
Nasp	5	81	ctgggattaggacagaaacatctggtgat (MSS4606)	cttctaaggcatttcccaacactccatt (MSS4607)
**Gene**	**Method**		**Forward primer (name)**	**Reverse primer (name)**
Mbnl1	G		ctacgatggctggctgcaatatgcctcactgtaag (MSS1382)	
Mbnl1	G			gggttgaatctcgttagggacactgggtgtctgtaa (MSS1383)
Mbnl1	G			cgtggcagaccctttgacaccgaatttc (MSS1901)
Mbnl2	G		gtaccaccttccttgtgatactgaaagctctgaggtc (MSS4254)	
Mbnl2	G		gtagggctctcaaggagagcactgcattgagc (MSS4255)	
Mbnl2	G			aatgtcaaaccagaccagaaatacaccaccatg (MSS4256)
Mbnl2	G		cgccttctatcgccttcttgacgagttcttctgag (MSS3365)	
Cre	G		tgcaacgagtgatgaggttcgcaagaacctg (MSS2684)	
Cre	G			gaacgaacctggtcgaaatcagtgcgttcg (MSS4412)
Mbnl2	q		gagtaattgcctgctttgattccctcaa (MSS4595)	
Mbnl2	q			ggagcggcgttcctggaaacata (MSS4596)
Gapdh	q		ggtgaaggtcggtgtgaacg (MSS4178)	
Gapdh	q			ctcgctcctggaagatggtg (MSS4179)

Abbreviations: nucleotides, nt; G, genotyping; q, qPCR
